# Impaired striatal glutathione–ascorbate metabolism induces transient dopamine increase and motor dysfunction

**DOI:** 10.1038/s42255-024-01155-z

**Published:** 2024-10-28

**Authors:** Mohd Yaseen Malik, Fei Guo, Aman Asif-Malik, Vasileios Eftychidis, Nikolaos Barkas, Elena Eliseeva, Kerstin N. Timm, Aleksandra Wolska, David Bergin, Barbara Zonta, Veronika Ratz-Wirsching, Stephan von Hörsten, Mark E. Walton, Peter J. Magill, Claus Nerlov, Liliana Minichiello

**Affiliations:** 1 Department of Pharmacology, University of Oxford, Oxford, UK; 2 MRC Molecular Haematology Unit, MRC Weatherall Institute of Molecular Medicine, University of Oxford and John Radcliffe Hospital, Oxford, UK; 3 MRC Brain Network Dynamics Unit, Nuffield Department of Clinical Neurosciences, University of Oxford, Oxford, UK; 4 Department of Experimental Therapy and Preclinical Centre, University Hospital and Friedrich-Alexander-University (FAU), Erlangen, Germany; 5 Department of Experimental Psychology, Oxford University, Oxford, UK; 6 Wellcome Centre for Integrative Neuroimaging, Oxford University, Oxford, UK

**Keywords:** Cellular neuroscience, Huntington's disease

## Abstract

Identifying initial triggering events in neurodegenerative disorders is critical to developing preventive therapies. In Huntington’s disease (HD), hyperdopaminergia—probably triggered by the dysfunction of the most affected neurons, indirect pathway spiny projection neurons (iSPNs)—is believed to induce hyperkinesia, an early stage HD symptom. However, how this change arises and contributes to HD pathogenesis is unclear. Here, we demonstrate that genetic disruption of iSPNs function by *Ntrk2/Trkb* deletion in mice results in increased striatal dopamine and midbrain dopaminergic neurons, preceding hyperkinetic dysfunction. Transcriptomic analysis of iSPNs at the pre-symptomatic stage showed de-regulation of metabolic pathways, including upregulation of *Gsto2*, encoding glutathione S-transferase omega-2 (GSTO2). Selectively reducing *Gsto2* in iSPNs in vivo effectively prevented dopaminergic dysfunction and halted the onset and progression of hyperkinetic symptoms. This study uncovers a functional link between altered iSPN BDNF-TrkB signalling, glutathione–ascorbate metabolism and hyperdopaminergic state, underscoring the vital role of GSTO2 in maintaining dopamine balance.

## Abstract (web summary)

Malik, Guo et al. show that Ntrk2/Trkb-mediated neurotrophic signalling regulates dopamine levels by controlling glutathione–ascorbate metabolism, thus impacting striatal dopaminergic circuits and motor function

## Main

Dopamine (DA) signalling in the striatum, a key integrator of cortical and thalamic information and a significant target of midbrain DA (mDA) neurons from the substantia nigra pars compacta (SNc)^1^, has an essential role in a wide range of behaviours, including control of voluntary motor movements, procedural learning, habit learning and cognition^1,2^. Dysfunctional DA transmission or signalling is found in many neurodegenerative disorders, including HD, an inherited neurodegenerative disorder caused by a highly polymorphic CAG trinucleotide repeat expansion in exon 1 of the Huntingtin gene (*HTT*). HD is characterized by adult-onset motor dysfunctions and cognitive decline^2^. Alterations in DA function have been linked to both motor and cognitive symptoms in HD. Post-mortem studies of brains from patients with HD have shown increased levels of DA and increased activity of tyrosine hydroxylase (TH), the rate-limiting enzyme in DA synthesis, in the striatum and SNc^3–5^. DA-depleting agents and DA receptor antagonists effectively reduced abnormal movements in patients with HD^3,4^, demonstrating the contribution of DA to motor dysfunction.

Several genetic models, including transgenic mice expressing mutant *HTT* (m*HTT*) fragments or full-length, m*HTT* knock-in mouse models^6^ and transgenic rat models^7^, were generated to reproduce the human condition and determine the cellular and molecular alterations underlying the pathogenesis of HD. Although each model differs in terms of CAG repeat length and transgene expression levels, and thus in the severity of the resulting HD phenotype, they were instrumental in demonstrating a fundamental feature of the disease, namely that severe neuronal dysfunction precedes degeneration and it is probably the primary cause of many HD symptoms^8,9^. For example, dysfunctional iSPNs, the most affected by HD, could initiate DA imbalance and, consequently, induce motor dysfunction. However, the cause of this change and its role in HD development are still not clear. Evidence from patients with HD and from rodent models implicates brain-derived neurotrophic factor (BDNF)-TrkB signalling deficiency in the pathogenesis of HD^10^. Yet dissecting the specific involvement of this signalling in models carrying m*HTT* is challenging, as m*HTT* causes various dysfunctions, including that of BDNF-TrkB signalling. Therefore, we previously focused on the most vulnerable neurons in HD, iSPNs, expressing enkephalin and D2 receptors (ENK^+^/D2R^+^)^11,12^. We demonstrated that TrkB signalling in these neurons is essential to maintain normal locomotor behaviour consistent with the initial hyperkinetic symptoms of HD and suggested that TrkB signalling deficiency may contribute effectively to HD motor symptoms^13^.

Here, we present compelling results demonstrating a functional connection between altered TrkB signalling in iSPNs and changes in glutathione metabolism, particularly the enzyme GSTO2, at the pre-symptomatic stage, causing an early increase in DA followed by progressive degeneration and the onset of hyperkinetic symptoms. This is consistent with decreased BDNF-TrkB signalling caused by m*HTT* both in patients with HD and in HD rodent models, driving early DA dysfunction and striatal vulnerability.

## Results

### Lack of TrkB signalling in iSPNs increases striatal DA

**Fig. 1:**
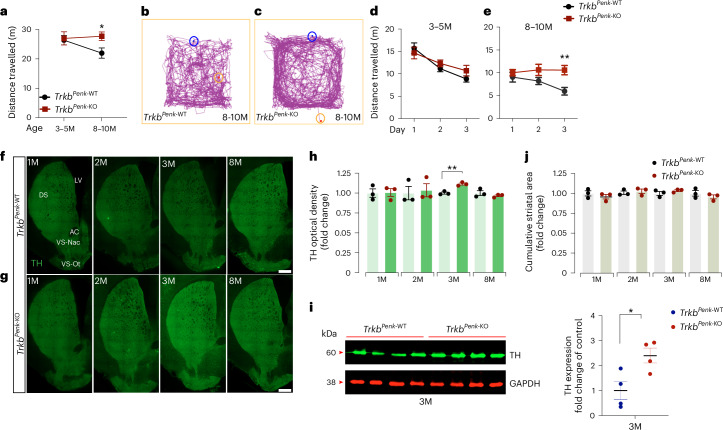
*Trkb*^*Penk*-KO^ mice develop hyperactivity following DA increase. **a**, Spontaneous locomotor activity from control and mutant female mice tested at two ages (*Trkb*^*Penk*^^-WT^, 3–5 months (*n* = 12), 8–10 months (*n* = 9); *Trkb*^*Penk*-KO^, 3–5 months (*n* = 11), 8–10 months (*n* = 9)). M, months. No difference was observed between groups at 3–5 months. A significant increase in total distance travelled was found in mutants at 8–10 months (*Trkb*^*Penk*-WT^, 21.97 ± 1.73 m vs *Trkb*^*Penk*-KO^, 27.71 ± 1.46 m; *P = 0.022). **b**,**c**, Representative tracks of control (**b**) and mutant (**c**) mice at 8–10 months showing the total distance travelled by the subject during the test time. Blue circle, beginning point; orange circle, endpoint. **d**,**e**, Habituation results for mice 3–5 months of age (main effect of time: *F*_(2,42)_ = 18.55, *P* < 0.0001; main effect of genotype: *F*_(1,21)_ = 0.4350, *P* = 0.51) (**d**) and 8–10 months of age (main effect of genotype: *F*_(1,16)_ = 9.804, *P* = 0.0064) and habituation (day 3, *Trkb*^*Penk*-WT^, 5.98 ± 0.84 m vs *Trkb*^*Penk-*KO^, 10.59 ± 1.02 m, **P = 0.0042) (**e**). **f**,**g**, Representative striatal TH-immunofluorescence images for *Trkb*^*Penk-*WT^ (**f**) and *Trkb*^*Penk-*KO^ (**g**) at different stages. **h**, Scatter–bar plot for TH expression levels determined by optical density fold change. Increased TH expression at 3 months in mutants (*Trkb*^*Penk-*WT^, 1 ± 0.0125 vs *Trkb*^*Penk-*KO^, 1.114 ± 0.0126, **P = 0.0031; no difference at other ages (1 month, *P* = 0.92; 2 months, *P* = 0.77, 8 months, *P* = 0.33), *n* = 3 each genotype and age analysed. A total of 12 females and 12 males were used in this experiment. **i**, Representative western blot from 3 month striatal tissues and a scatter–bar plot showing the quantification of TH protein levels expressed as fold change of control (*Trkb*^*Penk-*WT^, 1.003 ± 0.357; *Trkb*^*Penk-*KO^, 2.403 ± 0.295; *n* = 4 each genotype (all male), *P* = 0.02). GAPDH, loading control. **j**, Scatter–bar plot showing the mean cumulative striatal area of TH-immunofluorescence (mutant vs control: 1 month, *P* = 0.405; 2 months, *P* = 0.619; 3 months, *P* = 0.282; 8 months, *P* = 0.365). Values are means; error bars, s.e.m. *P* values in **a** from multiple unpaired *t*-tests (Holm–Šídák method); in **d** and **e** from two-way ANOVA and Šídák multiple comparison tests; in **h**, **i** and **j** from unpaired, two-tailed Student’s *t*-test. Scale bars in **f**–**g**, 500 μm. DS, dorsal striatum; VS-Nac, ventral striatum-nucleus accumbens; VS-Ot, ventral striatum-olfactory tubercle; LV, lateral ventricle. Source data

**Extended Data Fig. 1:**
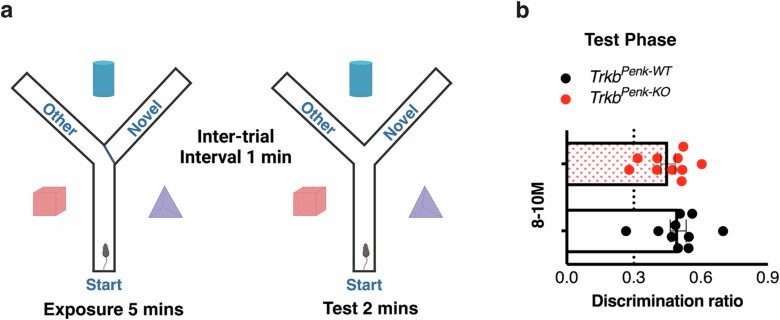
*Trkb*^*Penk-KO*^ mice display normal spatial novelty preference. **a**) Spontaneous spatial novelty preference was assessed using a 3-arm Y-maze enriched with distal cues. During the exposure phase, mice were assigned to two arms (the “start arm” and “other arm”) while the entrance to the third arm of the maze (the “novel arm”) was blocked off. Allocation of arms (start, other, and novel) to specific spatial locations was counterbalanced within each experimental group. Mice were placed at the end of the start arm and allowed to explore the start arm and the other for 5 min. Entry into an arm was defined as when a mouse placed all four paws into an arm, and similarly, a mouse was considered to have left an arm if all four paws were placed outside that arm. The mouse was removed from the maze and returned to its home cage for 1 min. During the test phase, mice were allowed access to all three arms of the maze and to explore for 2 min. The time spent by the mouse in each arm of the maze and the number of entries into each arm are recorded during the exposure and test phases. During the test phase, the preference for the novel arm was determined using a discrimination ratio (DR), which is calculated as the time spent exploring the novel arm divided by the total time spent in the start, familiar, and novel arms [novel/(novel + start+ familiar)]. During the exposure phase, mutants and controls explored both arms similarly (*p* = 0.70). **b**) During the test phase, the mutant and control groups showed a similar significant preference for the novel (previously unvisited) arm. Statistical analysis of the discrimination ratios for *Trkb*^*Penk-WT*^, 0.4987 ± 0.03532; *Trkb*^*Penk-KO*^, 0.4531 ± 0.03182; *p* = 0.35. Values are means ± SEM; *p* statistic from unpaired, two-tailed, Student’s t-test; n = 10 for each genotype (female mice). Related to Fig. 1. This figure was partially created with BioRender.com (the Y-maze). Source data

The cause of the DA dysfunction in patients with HD and in rodent models is currently unclear. Nevertheless, it may be a loss of neuroinhibitory control by striatal GABA^3^. Depletion of TrkB signalling in iSPNs impairs their inhibitory function, leading to spontaneous hyperactivity in *Trkb*^*Penk*-KO^ mice with increasing age^13^. Therefore, we asked whether TrkB signalling deficiency in iSPNs could trigger DA dysfunction. Increased DA levels in rodent models lacking the DA transporter are linked to typical disturbances like spontaneous hyperlocomotion and altered habituation to the testing environment^14^. We observed similar behaviour in *Trkb*^*Penk*-KO^ mice: from 8–10 months of age, mutants travelled significantly more than controls (*Trkb*^*Penk*-WT^) (Fig. 1a–c and ref. ^13^). Upon initial exposure to the open field, mice were re-exposed to the same arena for three consecutive days. Although 3–5 month-old mutants and controls habituated similarly, 8–10 month-old mutants showed an altered response to the testing environment and were unable to habituate (Fig. 1d,e). We previously established that *Trkb*^*Penk*-KO^ mice do not exhibit increased anxiety or heightened general activity^13^. To further investigate these findings, we determined whether the mutants exhibited an increased response to novelty. We performed a spontaneous spatial novelty preference test using a three-arm Y-maze alongside observing heightened motor activity in mice aged 8–10 months. During the test phase, mutant and control mice exhibited a similar preference for the novel arm, indicating no enhanced response to novelty in mutants (Extended Data Fig. 1a,b).

We then investigated the dopaminergic system at pre-symptomatic and symptomatic stages. Initially, we performed striatal immunofluorescence to analyse striatal TH expression from 1–8 months (Fig. 1f,g). TH expression was significantly higher in mutants at 3 months (Fig. 1f–h), confirmed by western blot analysis (Fig. 1i). At 8 months, the difference was no longer evident (Fig. 1h). The striatal TH-immunofluorescence cumulative area was similar between mutants and controls at all ages analysed (Fig. 1j), ruling out striatal shrinkage as the cause of increased TH.

**Fig. 2:**
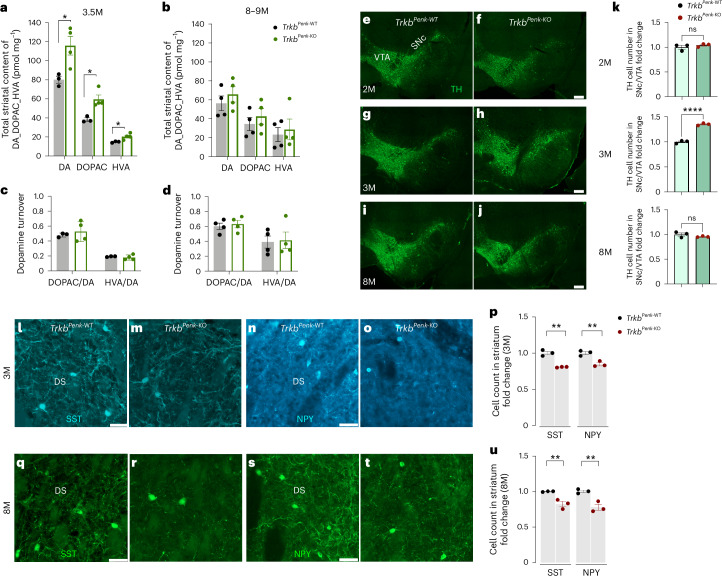
Changes in the SNc–VTA regions cause increased striatal DA. **a,b**, HPLC analysis of total tissue content of DA and its metabolites presented as scatter–bar plots. At 3.5 months of age (DA, *Trkb*^*Penk-*WT^, 80.47 ± 3.68 (*n* = 3) vs *Trkb*^*Penk-*KO^, 116.0 ± 9.73 (*n* = 4), *P = 0.030; DOPAC, *Trkb*^*Penk-*WT^, 38.96 ± 1.78 (*n* = 3) vs *Trkb*^*Penk-*KO^, 60.07 ± 4.53 (*n* = 4), *P = 0.012; HVA, *Trkb*^*Penk-*WT^, 15.61 ± 0.48 (*n* = 3) vs *Trkb*^*Penk-*KO^, 21.28 ± 1.59 (*n* = 4), *P = 0.032)) (**a**). At 8–9 months (*Trkb*^*Penk-*WT^ vs *Trkb*^*Penk-*KO^, DA, *P* = 0.43; DOPAC, *P* = 0.48; HVA, *P* = 0.69; *n* = 4 each group) (**b**), even though DA (16.5%), DOPAC (23%) and HVA (22%) were still higher in mutants compared with controls. All animals in this experiment were females. **c**,**d**, Normal DA turnover, DOPAC/DA and HVA/DA ratios at 3.5 months (**c**) and 8–9 months (**d**) showed similar ratios (*Trkb*^*Penk-*WT^ vs *Trkb*^*Penk-*KO^, DOPAC/DA, at 3.5 months, *P* = 0.57, and 8–9 months, *P* = 0.66. HVA/DA, at 3.5 months, *P* = 0.58, and at 8–9 months, *P* = 0.87. **e**–**j**, SNc–VTA representative images of TH-immunofluorescence for *Trkb*^*Penk-*WT^ and *Trkb*^*Penk-*KO^ at different ages (2 months (**e**,**f**), 3 months (**g**,**h**) and 8 months (**i**,**j**)). **k**, Scatter–bar plots showing TH^+^ cell counting with 35% increase in mutants at 3 months (*Trkb*^*Penk-*WT^, 6,417 ± 108.5 vs *Trkb*^*Penk-*KO^, 8,643 ± 80.96; ****P < 0.0001), but not earlier (2 months, 4% increase, *P* = 0.38) or later (8 months, −4%, *P* = 0.263), *n* = 3 each genotype and age, total of 12 females and 6 males all paired. **l**–**u**, Striatal SST^+^ and NPY^+^ cells analysis at ages of 3 months (all males) and 8 months (all males); representative immunofluorescence images from the dorsal striatum for SST (**l**,**m**) and NPY (**n**,**o**) at 3 months and for SST (**q**,**r**) and NPY (**s**,**t**) at 8 months. **p,u**, Scatter–bar plots show fold change comparison of SST^+^ and NPY^+^ cell number at 3 months (**p**) and 8 months (**u**). NPY (3 months, **P = 0.0012; 8 months, **P = 0.0097) *n* = 3 biological replicates for each genotype or group and age. SST (3 months, **P = 0.0071; 8 months, **P = 0.0083)   *n* = 3 biological replicates for each genotype or group and age. Values are means; error bars, s.e.m. *P* statistic in **a**, **b**, **c**, **d**, **k**, **p** and **u** from unpaired, two-tailed Student’s *t*-test. Scale bars in **e**–**j**, 200 μm; in **l**–**t**, 50 μm. VTA, ventral tegmental area; SNc, substantia nigra pars compacta; DS, dorsal striatum. Source data

**Extended Data Fig. 2:**
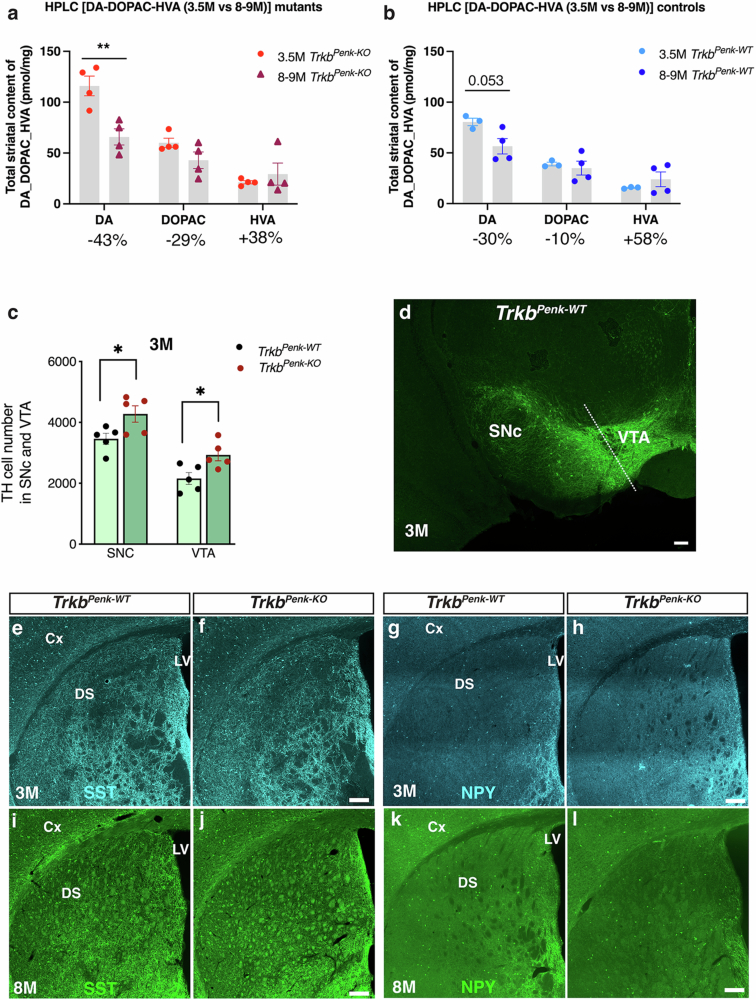
Dopamine imbalance and its impact on SST and NPY expression. **a**, **b**) Total dopamine content in the striatum of *Trkb*^*Penk-KO*^ decreases significantly by 8–9 months of age (−43%) after the initial increase at around three months (DA, 3.5 M vs 8–9 M, *p* = 0.007). However, DOPAC (−29%) and HVA (+38%) do not differ significantly between 3.5 M and 8–9 M (*p* = 0.1; *p* = 0.5, respectively). In controls, there is a slight decrease between 3.5 M and 8–9 M of age for DA content (−30%), *p* = 0.053; DOPAC (−10%), *p* = 0.64; and HVA (+58%), *p* = 0.37. Samples analysed are from Fig. 2a-b; 3.5 M, *Trkb*^*Penk-WT*^, n = 3; *Trkb*^*Penk-KO*^, n = 4. At 8–9 M, n = 4 for each group/genotype. **c**, **d**) Quantitative analysis of immunoreactive TH cell numbers in the SNc and VTA regions at 3 M. **c**) TH cell numbers in mutants indicate a significant increase in the SNc (*Trkb*^*Penk-WT*^, 3462 ± 182; *Trkb*^*Penk-KO*^, 4277 ± 269; *p = 0.036) as well as in the VTA (*Trkb*^*Penk-WT*^, 2156 ± 194.3; *Trkb*^*Penk-KO*^, 2932.6 ± 195.8; *p = 0.022); n = 5 each genotype, 3 females and 2 males. **d**) A representative epifluorescence image of a coronal section through the ventral midbrain shows TH immunofluorescence (green) in the SNc and VTA of *Trkb*^*Penk-WT*^ mice. The SNc/VTA distinction was made following Allen’s mouse brain atlas. **e**–**l**) Representative IF images for SST and NPY at 3 M and 8 M of the whole dorsal striatum (**SST**, panels **e**, **f** and **i**, **j**; **NPY**, panels **g**, **h** and **k**, **l**) of *Trkb*^*Penk-WT*^ and *Trkb*^*Penk-KO*^. Scale bars, **f**, **j** and **h**, **l**, 200μm; **d**, 50μm. VTA, ventral tegmental area; SNc, substantia nigra pars compacta. (**a**–**c**) Values are means ± SEM. *p* statistic from unpaired, two-tailed, Student’s t-test. Related to Fig. 2. Source data

We estimated the total striatal tissue content of DA and its metabolites using high-performance liquid chromatography (HPLC)-coupled electrochemical analysis. At 3.5 months, mutant samples exhibited a significant 44% increase in DA levels along with a higher concentration of DA primary metabolites, specifically 3,4-dihydroxyphenylacetic acid (DOPAC) at 54% and homovanillic acid (HVA) at 36% (Fig. 2a). This increase was no longer significant at 8–9 months (Fig. 2b). DA, DOPAC and HVA content did not change significantly in controls with age. By contrast, a significant decrease in DA content but not DOPAC or HVA was observed in mutants (Extended Data Fig. 2a,b). DA turnover was similar between genotypes at both ages analysed (Fig. 2c,d).

We then asked whether changes in the SNc and ventral tegmental area (VTA) regions, the primary sources of striatal DA, led to elevated striatal TH expression and DA levels. We conducted a time-course analysis to determine the number of TH^+^ cells in the SNc–VTA regions of the mutant and control subjects. Quantitative analysis showed a significant increase in TH^+^ cells at 3 months in *Trkb*^*Penk*-KO^ SNc–VTA regions, whether analysed as a whole or separately (Fig. 2e–k and Extended Data Fig. 2c,d). Thus, increased striatal TH expression at 3 months in *Trkb*^*Penk*-KO^ mice was caused by a rise in TH^+^ cell number in the SNc–VTA regions. This increase was not observed at 2 months or later at 8 months (Fig. 2k), consistent with the findings in the striatum (Fig. 1f–h).

Ample evidence suggests that an imbalance in dopaminergic tone impacts the expression levels of neuropeptides like somatostatin (SST) and neuropeptide Y (NPY), as seen in patients with Parkinson’s disease and HD as well as in rodent models^15–18^. Therefore, to support our findings, we investigated the impact of increased DA in *Trkb*^*Penk*-KO^ on striatal NPY and SST. A significant decrease in immunoreactive NPY^+^ (−19%) and SST^+^ (−15%) neuron numbers was observed at 3 months in the striatal tissues of mutants (Fig. 2l–p and Extended Data Fig. 2e–h). Interestingly, such decrease persisted at 8 months (NPY, −17%; SST, −22%) (Fig. 2q–u and Extended Data Fig. 2i–l), although DA was decreased to control levels at that stage. This finding supports the idea that the initial striatal hyperdopaminergia in *Trkb*^*Penk*-KO^ causes long-lasting cellular and molecular changes that induce spontaneous hyperlocomotion.

### Unexpected changes discovered in the pre-symptomatic phase

**Extended Data Fig. 3:**
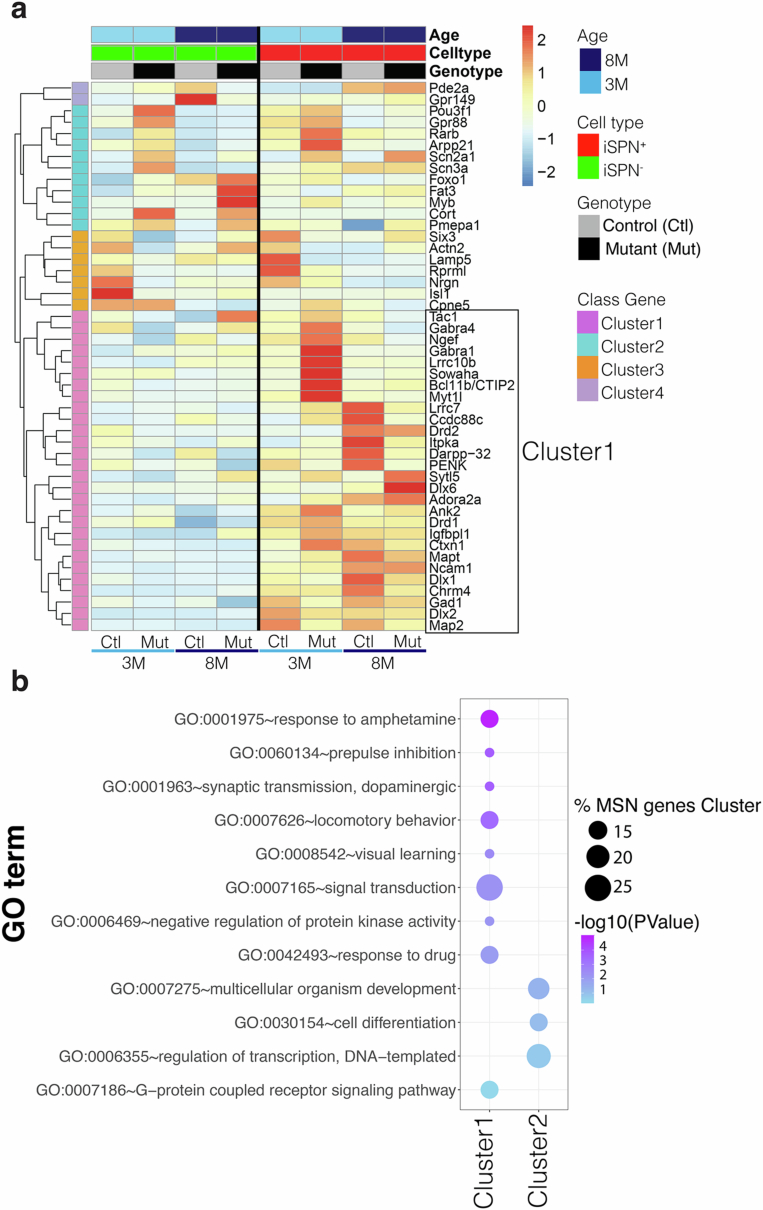
Significant enrichment of iSPN signature gene expression in sorted striatal subpopulations. **a**) Heatmap representation of average expression values of 48 SPN marker genes^23^ in the different sorted striatal populations. The black frame highlights the largest cluster (n.1). **b**) Bubble plot visualising DAVID gene ontology (GO) analysis using the enriched gene set from cluster 1/2 in (**a**). Significant enrichment of GO terms associated with critical functions of iSPN+ is shown as a colour gradient with highly significant enriched terms in purple and low ones in sky blue. The bubble size indicates the percentage of gene clusters. Related to Fig. 3.

**Extended Data Fig. 4:**
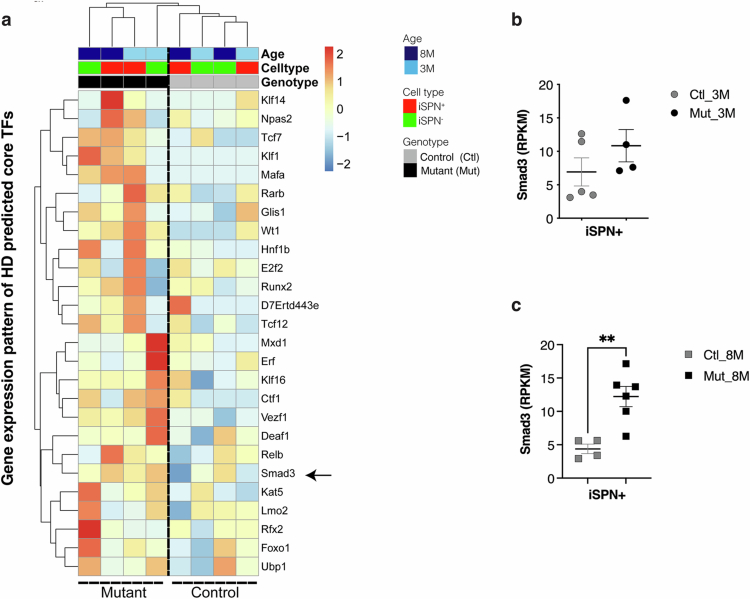
Significant enrichment of HD core transcription factors in iSPN mutant subpopulation. **a**) Heatmap displaying the enrichment of 26 HD core TFs among different striatal purified populations at 3 M and 8 M. **b**, **c**) *Smad3* gene expression levels in iSPN^+^ at 3 M (Control vs Mutant, *p* = 0.2) and 8 M (Control vs Mutant, *p* = 0.004 **). Values are means ± SEM; *p* statistic from unpaired, two-tailed Student’s t-test. Related to Fig. 3. Source data

To identify the cellular and molecular changes induced by neurotrophin signalling deficiency, increased DA and ageing that may promote iSPN vulnerability, we examined the transcriptional profile of relevant neurons in the presence and absence of TrkB signalling at 3 months and 8 months. We optimized the Smart-Seq2 (ref. ^19^) method to profile adult and aged central nervous system neuronal populations from as few as 200 neurons per mouse^20^. Therefore, neuronal subsets (iSPNs) from mouse striatal tissues of mutants and controls (here called iSPN^+^mut and iSPN^+^wt populations, respectively) were genetically labelled in vivo with a fluorescent marker using Rosa26-Ai9-tdTomato strain^21^ and purified by fluorescence-activated cell sorting. At the same time, we purified tdTomato-negative striatal cells (which include any other cell type in the striatal tissue) from both controls and mutants (here called iSPN^−^wt and iSPN^−^mut, respectively). After initial quality-control steps and data filtering, we used hierarchical clustering to identify gene expression patterns. Initially, we confirmed the SPN signature by comparing the gene expression profiles of the sorted striatal populations with known genes highly enriched in the striatum^22^. This analysis revealed that 28 out of 48 genes (cluster 1), including *Penk*, *Adora2a, Drd2* and *Darpp32*, were highly enriched in iSPN^+^ populations at 3 months and 8 months compared with the iSPN^−^ populations. However, the iSPN^+^mut population at 3 months had already impacted numerous marker genes (Extended Data Fig. 3a). Gene ontology analysis of the gene set from cluster 1 revealed a significant enrichment of gene ontology terms associated with critical cellular processes, including dopaminergic synaptic transmission, prepulse inhibition and enriched biological processes (like locomotor behaviour and drug response) (Extended Data Fig. 3b). In addition, hierarchical clustering revealed 26 core transcription factors predicted to be key driver genes of the HD progression^23^ enriched particularly in the 3 month iSPN^+^mut population (Extended Data Fig. 4a). Notably, *Smad3*, which acts primarily downstream of the TGF-β–activin pathway and is predicted to drive early gene expression changes in HD^23^, was significantly upregulated in the iSPN^+^mut subpopulation at 8 months (Extended Data Fig. 4b), suggesting that a deficiency in TrkB signalling in iSPN^+^ neurons may contribute to the transcriptional dysregulation observed in the HD striatal region.

**Fig. 3:**
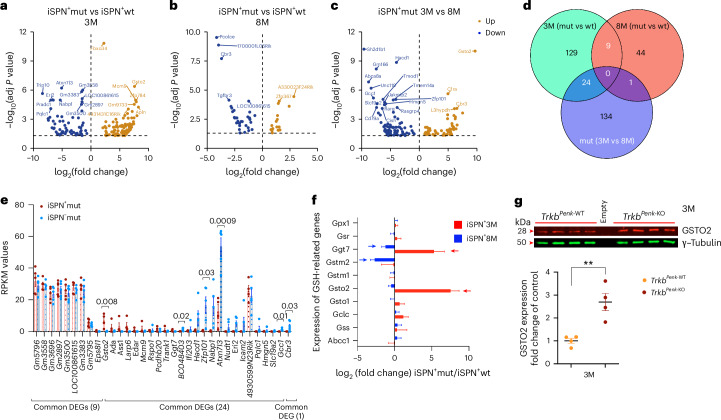
Transcriptional changes induced by *Trkb* deletion in iSPN. **a**–**c**, Volcano plots summarizing the RNA-seq data results. DEGs between iSPN^+^mut and control at 3 months (**a**) and 8 months (**b**) and from the comparison between iSPN^+^mut 3 months vs 8 months (**c**). Brown and blue dots indicate genes with significantly increased or decreased expression, respectively. Genes with log_10_(adj *P* value) > 4 are indicated in **a**–**c**. **d**, Venn diagram representing unique and common genes differentially expressed in iSPN^+^ population comparison sets. **e**, Comparison of RPKM values between iSPN^+^mut populations and the respective counterpart (iSPN^−^mut populations) for the 34 overlapping genes from the Venn diagram in **d**. At 8 months, comparing iSPN^+^mut and iSPN^-^mut populations (*n* = 6 biological replicas per population type), *Cbr3* was specifically downregulated in iSPN^+^mut vs iSPN^−^mut (*P* = 0.03). At 3 months, iSPN^+^mut populations (*n* = 4 biological replicas) and iSPN^−^mut populations (*n* = 5 biological replicas), four genes (*Gcc1*, *P* = 0.01; *Atxn7l3*, *P* = 0.0009; *Zfp101*, *P* = 0.03, and *BC048403*, *P* = 0.02) were specifically downregulated whereas *Gsto2* (*P* = 0.008) was specifically upregulated in iSPN^+^mut vs iSPN^−^mut. RPKM, reads per kilobase of transcript per million reads mapped. **f**, Plot showing some DEGs (DESeq2) involved in the glutathione metabolism (log_2_(fold change) of iSPN^+^mut/iSPN^+^wt). At 3 months (iSPN^+^mut, *n* = 5 and iSPN^+^wt, *n* = 5 biological replicas), genes were significantly upregulated (red arrow) (*Gsto2*, adj *P* = 4.2 × 10^−7^; *Ggt7*, adj *P* = 0.02) or downregulated (blue arrow) at 8 months, (iSPN^+^mut, *n* = 6 and iSPN^+^wt, *n* = 4 biological replicas), *Gstm2* (adj *P* = 0.0007) and *Ggt7* (adj *P* = 0.01). **g**, Representative western blot for 3 month striatal tissues lysate and quantification of GSTO2 protein levels expressed as fold change of control in a scatter plot (*Trkb*^*Penk-*WT^, 1.0 ± 0.114; *Trkb*^*Penk-*KO^, 2.698 ± 0.376, *n* = 4 of each genotype, all male mice, **P = 0.005). γ-Tubulin, loading control. Values are means; error bars, s.e.m. In **a**–**d**, *n* = 4–6 biological replicates per genotype or age; all male mice; *P* statistic from unpaired *t*-tests adjusting the *P* value with the false discovery rate method (two-stage step-up Benjamini, Krieger and Yekutieli). In **g**, *P* statistic from unpaired, two-tailed Student’s *t*-test. Source data

We then used the DESeq2 method^24^ to analyse count data differentially. There were 162 differentially expressed genes (DEGs) at 3 months and only 54 DEGs at 8 months in iSPN^+^mut compared with iSPN^+^wt (Fig. 3a,b). Comparing iSPN^+^mut 3 month vs 8 month populations identified 159 DEGs specifically dysregulated in the iSPN^+^mut population at 3 months (Fig. 3c). Examining unique and common DEGs in comparison sets of iSPN^+^ populations revealed a total of 34 common genes (Fig. 3d). Finally, to confirm the cell-type-specific dysregulation of these 34 overlapping genes, we compared their mRNA expression levels (RPKM) with those of their respective counterpart (iSPN^−^mut population) (Fig. 3e). At 3 months, we observed four genes (*Gcc1*, *Atxn7l3*, *Zfp101* and *Bc048403*) that were specifically downregulated and one (*Gsto2*) that was specifically upregulated in the iSPNs^+^mut compared with the iSPN^−^mut populations (Fig. 3e). At 8 months, carbonyl reductase (*Cbr3*) was downregulated in the iSPN^+^mut population (Fig. 3e). These results indicated significant and distinct alterations in iSPN^+^mut neurons at the pre-symptomatic stage. The *Gsto2*-specific upregulation at 3 months was the most interesting result. The glutathione S-transferase (GST) superfamily comprises several independent members, including the omega (*Gsto1* and *Gsto2*) and the mu (*Gstm1* and *Gstm2*) families. These enzymes are involved in detoxification processes^25^ by linking toxic compounds with glutathione (GSH), a key antioxidant non-protein thiol, thus counteracting oxidative stress^26^. Altered GST enzyme levels and/or function have been proposed to contribute to disease susceptibility in neurodegenerative diseases^27^. Genetic linkage studies have discovered associations between GSTO genes and age-at-onset of Alzheimer’s disease, Parkinson’s disease and amyotrophic lateral sclerosis^28,29^. Nonetheless, there is limited knowledge about GSTOs and their impact on striatal neurons in polyQ diseases like HD. In our model, the absence of TrkB signalling in iSPN^+^ led to specific upregulation of *Gsto2* at the pre-symptomatic stage, followed by a sharp decrease to baseline levels by 8 months (Fig. 3f). The *Gsto2* upregulation was also confirmed by western blot analysis (Fig. 3g). In addition, gamma-glutamyltransferase-7 (*Ggt7*)^30^, which maintains GSH balance, demonstrated a biphasic pattern in iSPN^+^mut similar to *Gsto2* (Fig. 3f). Among other genes involved in GSH metabolism, glutathione transferase M2-2 (*Gstm2*), which is suggested to prevent the neurotoxic effects of DA oxidation on dopaminergic neurodegeneration in Parkinson’s disease^31^, was significantly downregulated by 8 months (Fig. 3f). Therefore, significant molecular changes during the pre-symptomatic phase caused by TrkB signalling depletion may induce iSPN vulnerability.

### Altered striatal energy metabolism at the pre-symptomatic phase

**Fig. 4:**
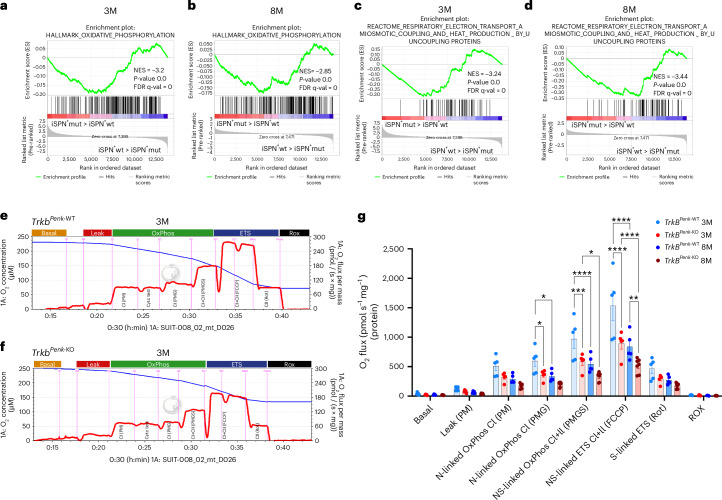
Energy metabolism is impaired in the striatal tissue of pre-symptomatic *Trkb*^*Penk-*KO^ mice. **a**,**b**, GSEA of iSPN^+^mut versus iSPN^+^wt at pre-symptomatic (3 months) (**a**) and symptomatic (8 months) (**b**) phases using the Molecular Signatures Database (MSigDB) H (hallmark) gene sets. **c**,**d**, GSEA of iSPN^+^mut versus iSPN^+^wt at 3 months (**d**) and 8 months (**e**) using C2 (curated) gene sets. *n* = 4–6 biological replicas per genotype and age (all male mice). GSEA statistics were computed by sorting each gene list in descending order of log_2_(fold change). The significance of gene sets was evaluated at a false discovery rate (FDR) of <0.05. Normalized enrichment score (NES), *P* value and FDR are indicated. **e**,**f**, Representative respirometry traces showing oxygen concentration (blue trace) and oxygen flux (oxygen consumption rate in pmol s^−1^ mg^−^^1^ tissue, red trace) assessed in striatum homogenates from *Trkb*^*Penk-*WT^ (**e**) and *Trkb*^*Penk-*KO^ (**f**) mice at 3 months with an O2k high-resolution respirometer. Different respiratory states were evaluated as follows: basal respiration; leak respiration after the addition of pyruvate and malate (PM) as respiratory substrates feeding into complex I (NADH or N-linked); N-linked OxPhos after addition of saturating concentrations of ADP (D); assessment of mitochondrial inner membrane integrity by addition of exogenous cytochrome *c*; ADP-stimulated N-linked OxPhos after additional supplementation with glutamate (G) as respiratory fuel; NS-linked OxPhos after addition of succinate (substrate for complex II); NS-linked ETS capacity rate with the uncoupler carbonyl cyanide-p-(trifluoromethoxy) phenylhydrazone (FCCP); S-linked ETS capacity rate after addition of the CI inhibitor rotenone (Rot); residual oxygen consumption (ROX) after addition of the complex III inhibitor antimycin A. **g**, Graphical representation by scatter–bar plot of the results obtained in **e** and **f** presented in pmol s^−1^ mg^−1^ protein. Different respiratory states in *Trkb*^*Penk-*WT^ and *Trkb*^*Penk-*KO^ mice at 3 months and 8 months age (*n* = 5 for controls and mutants at 3 months (all male mice), *n* = 5 for control and *n* = 7 for mutants at 8 months (males and females) were analysed as described in **e** and **f**. Values are means, error bars, s.e.m. Significant differences observed by two-way ANOVA and Tukey’s multiple comparisons tests are indicated by asterisks (*P ≤ 0.05; **P ≤ 0.01, ***P = 0.0003, ****P ≤ 0.0001) and are detailed in Table S2. There was no significant difference between the two genotypes at 3 months or 8 months in the basal O_2_ consumption rates (*P* > 0.05), ROX respiratory state (*P* > 0.05) or OxPhos coupling efficiency (*P* > 0.05). Source data

To better understand how TrkB signalling depletion affects changes in iSPN gene expression, we conducted gene set enrichment analysis (GSEA)^32^. We identified highly deregulated pathways in iSPN^+^mut, with changes already apparent at 3 months. As expected by the increased striatal DA, pathways signifying activation of signalling by G-protein-coupled receptors, transmission across chemical synapses and calcium signalling were all positively enriched at 3 months in iSPN^+^mut (Supplementary Table 1a), whereas genes associated with metabolic pathways such as oxidative phosphorylation (OxPhos) and respiratory electron transport ATP synthesis were significantly downregulated in iSPN^+^mut compared to iSPN+wt at 3 months (Fig. 4a,c and Supplementary Table 1b,c), followed by a further significant decline at 8 months (Fig. 4b,d and Supplementary Table 1e,f). Therefore, the absence of TrkB signalling in iSPN^+^ triggers severe energy deficits, possibly caused by mitochondrial dysfunction that suppresses OxPhos processes.

To investigate this possibility, we used high-resolution respirometry and analysed various respiratory states in the striatal tissues of mutants and controls at 3 months and 8 months. The findings supported the GSEA results and identified specific affected respiratory states (Fig. 4e–g). There was a significant decrease in three main respiratory states: NADH (N)-linked OxPhos, NADH-succinate (NS)-linked OxPhos and NS-linked electron transfer system (ETS) (maximum uncoupled electron transfer capacity). OxPhos is achieved in the presence of saturating ADP levels and the sequential addition of different mitochondrial substrates, including pyruvate, malate, glutamate (N-linked, through complex I) and succinate (S-linked, through complex II). Both N-linked and S-linked OxPhos, as well as NS-linked ETS capacity rate after the addition of the uncoupler carbonyl cyanide-p-(trifluoromethoxy) phenylhydrazone (FCCP), were significantly reduced in mutants compared with controls at 3 months (Fig. 4g). At 8 months, only the NS-linked ETS capacity rate was further significantly reduced in mutants, as both N-linked and NS-linked OxPhos were already significantly reduced with age in controls (Fig. 4g and Supplementary Table 2). Comparison between 3 month and 8 month *Trkb*^*Penk*-KO^ mutants revealed a further reduction of the NS-linked OxPhos rate and the NS-linked ETS capacity rate (Fig. 4g and Supplementary Table 2). None of the samples had a cytochrome *c* test exceeding 10%, suggesting mitochondrial inner membrane integrity. Collectively, these data uncovered a severe mitochondrial OxPhos dysfunction in pre-symptomatic *Trkb*^*Penk*-KO^ mice, consistent with the RNA sequencing (RNA-seq) data and GSEA pathway analysis. Additionally, GSEA analysis of iSPN^+^mut at 3 months revealed a significant reduction in the expression of genes associated with the first stage of the cellular respiration process glycolysis and the tricarboxylic acid cycle, followed by a further decrease at 8 months (Supplementary Table 1b,c,e,f). Thus, increased DA and early changes in energy metabolism impact neuronal viability before symptom onset in *Trkb*^*Penk*-KO^ mice.

### Glutathione metabolism is altered before symptoms appear

**Extended Data Fig. 5:**
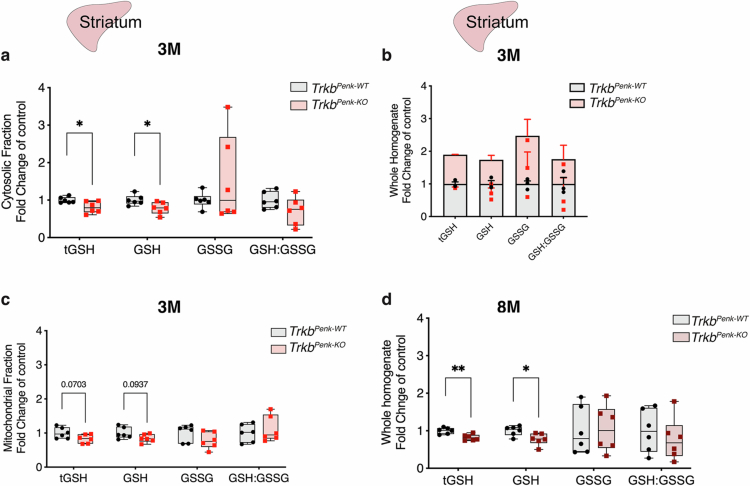
Striatal Glutathione levels and redox status are impaired in *Trkb*^*Penk-KO*^ mutants. **a**, **b**) Concentration of total (tGSH) and reduced (GSH) glutathione in striatal tissues of *Trkb*^*Penk-KO*^ and control littermates at 3 M and 8 M of age. The measurement is expressed as nmol/mg protein and presented as a fold change. **a**) Data are presented as box plots min-max (median), cytosolic fraction at 3 M, tGSH (*Trkb*^*Penk-WT*^ 1.0 ± 0.03; *Trkb*^*Penk-KO*^, 0.81 ± 0.06, *p* = 0.02); GSH (*Trkb*^*Penk-WT*^, 1:00 ± 0.05; *Trkb*^*Penk-KO*^ 0.78 ± 0.06, *p* = 0.02); GSSG (*Trkb*^*Penk-WT*^, 1:00 ± 0.08; *Trkb*^*Penk-KO*^, 1.54 ± 0.48, *p* = *0.29*) and GSH:GSSG (*Trkb*^*Penk-WT*^, 1:00 ± 0.09; *Trkb*^*Penk-KO*^, 0.71 ± 0.15, *p* = *0.13*). **b**) Whole homogenate at 3 M, tGSH (*Trkb*^*Penk-WT*^, 1:00 ± 0.06; *Trkb*^*Penk-KO*^, 0.89 ± 0.01, *p* = *0.16*); GSH (*Trkb*^*Penk-WT*^, 1:00 ± 0.10; *Trkb*^*Penk-KO*^, 0.74 ± 0.14, *p* = *0.14*); GSSG (*Trkb*^*Penk-WT*^, 1:00 ± 0.09; *Trkb*^*Penk-KO*^, 1.48 ± 0.50, *p* = *0.50*) and GSH:GSSG (*Trkb*^*Penk-WT*^, 1:00 ± 0.20; *Trkb*^*Penk-KO*^, 0.76 ± 0.43, *p* = *0.63*). **c**) Data are presented as box plots min-max (median), mitochondrial fraction at 3 M, tGSH (*Trkb*^*Penk-WT*^, 1:00 ± 0.07; *Trkb*^*Penk-KO*^, 0.83 ± 0.05, *p* = *0.07*); GSH (*Trkb*^*Penk-WT*^, 1:00 ± 0.07; *Trkb*^*Penk-KO*^, 0.84 ± 0.05, *p* = *0.09*); GSSG (*Trkb*^*Penk-WT*^, 1:00 ± 0.10; *Trkb*^*Penk-KO*^, 0.78 ± 0.10, *p* = *0.15*) and GSH:GSSG (*Trkb*^*Penk-WT*^, 1:00 ± 0.11; *Trkb*^*Penk-KO*^, 1.12 ± 0.16, *p* = *0.55*). **d**) Whole homogenate at 8 M, data are presented as box plots min-max (median), tGSH (*Trkb*^*Penk-WT*^, 1:00 ± 0.03; *Trkb*^*Penk-KO*^, 0.82 ± 0.03, *p* = 0.003); GSH (*Trkb*^*Penk-WT*^, 1:00 ± 0.05; *Trkb*^*Penk-KO*^, 0.77 ± 0.07, *p* = 0.02); GSSG (*Trkb*^*Penk-WT*^, 1:00 ± 0.26; *Trkb*^*Penk-KO*^, 1.06 ± 0.25, *p* = *0.87*) and GSH:GSSG (*Trkb*^*Penk-WT*^, 1:00 ± 0.23; *Trkb*^*Penk-KO*^, 0.77 ± 0.23, *p* = *0.50*). Values are means ± SEM; *p* statistic from unpaired, two-tailed, Student’s t-test. Animals, **a**, **c**, **d**) n = 6 each genotype (**a**, **c**, female mice; **d**, male mice. **b**) n = 3 each genotype (male mice). Box plot statistics for panels **c**, **e**, and **f** are reported in the source data. Source data

In our model, altered energy metabolism at 3 months was concomitant with altered glutathione metabolism selectively in the iSPN^+^mut. There was evidence for glutathione oxidation during the reduction of dehydroascorbate (DHA) involving GSTO2 (ref. ^33^) but also an altered GSH conjugation pathway, an important cell detoxification mechanism, showing significantly increased expression of genes associated with this pathway (15 genes out of 36 were affected; Supplementary Table 2a). Similarly, the DA increase observed at 3 months was no longer significant in mutants compared with controls at 8 months, when apoptosis pathways increased (Supplementary Table 1d). Although no cell death was detected in striatal tissues at this age^13^, changes in gene expression suggest a degenerative process in these neurons. GSH is a tripeptide (glutamic acid, cysteine and glycine). The cysteinyl thiol is a potent reducing agent, making GSH the most abundant antioxidant intracellular small molecule thiol. Within cells, GSH exists in a balanced ratio of the reduced sulfhydryl form (GSH) and the oxidized glutathione disulfide form (GSSG). Its intracellular concentration usually indicates oxidative cellular stress, as the latter profoundly affects the cellular thiol balance, usually causing a decrease in the cellular GSH/GSSG ratio. Following a previous publication^34^, we used the enzymatic recycling method to determine GSH concentration and GSSG levels in the striatal tissues of mutants and controls at two stages. At 3 months, the total glutathione (tGSH), GSH and GSSG levels in the whole homogenate did not significantly differ between mutants and controls. Upon cellular fractionation, mutants exhibited a significant decrease in tGSH and GSH in the cytosolic fraction compared to controls, where most cellular GSH is located (Extended Data Fig. 5a,b). The mitochondrial fraction did not differ (Extended Data Fig. 5c). At 8 months, tGSH and GSH from the whole homogenate were significantly decreased in mutants compared with controls (Extended Data Fig. 5d), suggesting disrupted GSH redox homeostasis in mutant striatal tissue.

### Altered striatal glutathione metabolism affects the dopaminergic system

**Fig. 5:**
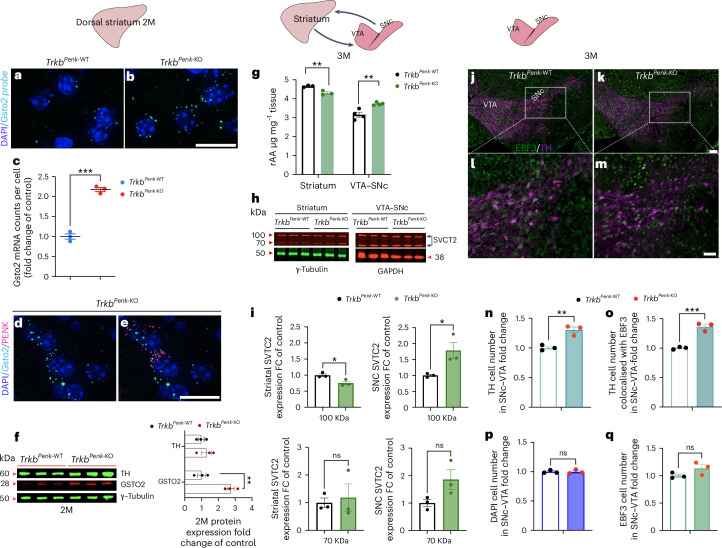
*Gsto2* increase in *Trkb*^*Penk*-KO^ iSPNs impacts DA levels via AA homeostasis. **a**,**b**, *Gsto2* upregulation in iSPNs precedes DA increase. Dorsal striatum representative *Gsto2* smFISH images from *Trkb*^*Penk-*WT^ (**a**) and *Trkb*^*Penk-*KO^ mice (**b**) at 2 months (see Methods and probe details in Supplementary Table 5). **c**, Scatter plot of *Gsto2* mRNA counts per cell comparing *Trkb*^*Penk-*KO^ with *Trkb*^*Penk-*WT^ mice. ***P < 0.0002, *n* = 3 per genotype (female mice). **d**,**e**, Representative *Gsto2* mRNA (smFISH) images colocalizing with PENK protein in iSPN of *Trkb*^*Penk-*KO^ dorsal striatum. **f**, Western blot for GSTO2 and TH expression analysis from striatal tissue homogenates of *Trkb*^*Penk-*KO^ vs *Trkb*^*Penk-*WT^ at 2 months and respective scatter–bar plot (GSTO2, *P* = 0.0052; TH, *P* = 0.41). γ-Tubulin, loading control; *n* = 6 (four males and two females paired). **g**, Scatter–bar plots of rAA concentrations measured by HPLC in striatal and midbrain dopaminergic area tissue homogenate at 3 months. Striatum (*Trkb*^*Penk-*WT^, 4.64 ± 0.026; *Trkb*^*Penk-*KO^, 4.29 ± 0.06, *P* = 0.0081, *n* = 3 per genotype, male mice); mDA (*Trkb*^*Penk-*WT^, 3.16 ± 0.126; *Trkb*^*Penk-*KO^, 3.72 ± 0.038, *P* = 0.005, *n* = 4 per genotype, male mice). **h**, Representative western blot images for the AA transporter, SVCT2. The 70 kDa and the 100 kDa forms are present in both regions, striatum and mDA area (SNc–VTA). **i**, Scatter–bar plot showing quantification of SVTC2 protein levels in striatal tissue (p100 kDa, *Trkb*^*Penk-*WT^, 0.99 ± 0.04; *Trkb*^*Penk-*KO^, 0.76 ± 0.05, *P* = 0.026; p70 kDa, *Trkb*^*Penk-*WT^, 1.0 ± 0.17; *Trkb*^*Penk-*KO^, 1.19 ± 0.49, *P* = 0.7) and in mDA tissue (*Trkb*^*Penk-*WT^, 1.0 ± 0.03; *Trkb*^*Penk-*KO^, 1.78 ± 0.25, *P* = 0.038; p70 kDa, *Trkb*^*Penk-*WT^, 1.0 ± 0.13; *Trkb*^*Penk-*KO^, 1.85 ± 0.35, *P* = 0.08). GAPDH and γ-Tubulin, loading control; *n* = 3 for each genotype (male mice). **j**,**k**, Representative SNc–VTA area images immunostained for TH and EBF3. **l**,**m**, Enlarged view of the images in **j** and **k**. **n**–**q**, Scatter–bar plots showing fold-change-specific cell counts in SNc–VTA area of mutants and controls. **n**, Scatter–bar plot showing TH^+^ cell number significant increase in 3 month mutants (*Trkb*^*Penk-*WT^, 1.0 ± 0.025 vs *Trkb*^*Penk-*KO^, 1.3 ± 0.05; **P = 0.005). **o**, Scatter–bar plot showing TH^+^/EBF3^+^ colocalized cell number (*Trkb*^*Penk-*WT^, 1.0 ± 0.012 vs *Trkb*^*Penk-*KO^, 1.36 ± 0.04; ***P = 0.001). **p**, Scatter–bar plot showing DAPI total cell number (*Trkb*^*Penk-*WT^, 1.0 ± 0.01 vs *Trkb*^*Penk-*KO^, 1.0 ± 0.02; *P* = 0.98). **q**, Scatter–bar plot showing EBF3^+^ cell number (*Trkb*^*Penk-*WT^, 1.0 ± 0.03 vs *Trkb*^*Penk*-KO^, 1.14 ± 0.065; *P* = 0.13). Values are means; error bars, s.e.m.; *n* = 3 for each genotype and age, male mice. **c,** **f,** **g,** **h,** **n**–**q**) *P* statistics are from unpaired, two-tailed, Student’s *t*-tests. Scale bars in, **a**,**b**,**d**,**e**, 20 μm; in **j**,**k**, 100 μm; in **l**,**m**, 50 μm. Source data

**Extended Data Fig. 6:**
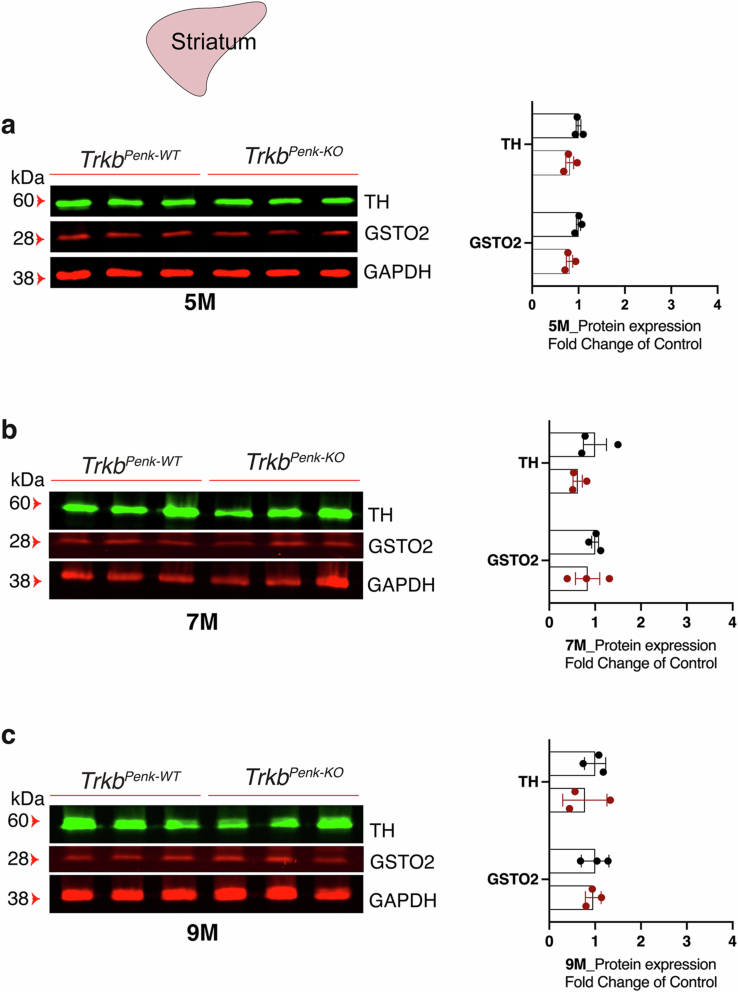
Time-course expression analysis for striatal GSTO2 and TH. **a**–**c**) Expression analysis of GSTO2 and TH by WB from striatal tissue homogenates of mutants and controls at different stages, with respective quantification in fold change of control. *Trkb*^*Penk-KO*^ *vs Trkb*^*Penk-WT*^ **a**) at 5 M (GSTO2, *p* = 0.076; TH, *p* = 0.12), n = 3 each cohort/genotype, (1 male and 2 females per cohort/genotype); **b**) at 7 M (GSTO2, *p* = 0.58; TH, *p* = 0.23), n = 3 each cohort/genotype, all females; **c**) at 9 M (GSTO2, *p* = 0.85; TH, *p* = 0.50), n = 3 each cohort/genotype, all females). GAPDH loading control; values are means ± SEM; *p* statistic from unpaired, two-tailed, Student’s t-test. Related to Fig. 5. Source data

A sustained increase in extracellular DA induces selective degeneration of its target neurons, SPNs^35^. In our model, DA shows time-dependent changes, with an initial increase at 3 months and a decrease to control levels by 8–9 months (Figs. 1 and 2). This pattern was also observed for genes in the glutathione metabolism pathway, particularly *Gsto2* in iSPNs. To determine whether the increase in DA was preceded by *Gsto2* upregulation, we conducted single-molecule fluorescent in situ hybridization (smFISH) analysis on striatal sections of mutants and control mice. At 2 months, the mutants exhibited a significant increase in *Gsto2* compared to the control group (Fig. 5a–c). Combining *Gsto2* smFISH with PENK immunofluorescence (Fig. 5d,e) confirmed increased *Gsto2* mRNA expression in enkephalinergic neurons, supporting the RNA-seq results (Fig. 3e). Further evidence was provided by a time-course western blot analysis of striatal GSTO2 and TH protein levels, showing GSTO2 upregulation before TH increase by 1 month (Figs. 5f and 1i), consistent with the immunofluorescence analysis of TH (Figs. 1f–i and 2e–k). The upregulation of GSTO2 and TH was followed by a decrease to control levels by 5 months of age. No further changes were observed at 7 months and 9 months (Extended Data Fig. 6a–c).

We then asked whether *Gsto2* triggers the dopaminergic dysfunction observed in *Trkb*^*Penk*-KO^ mice. Intriguingly, GSTO2 has exceptionally high dehydroascorbate reductase (DHAR) activity and, therefore, it may help maintain adequate levels of ascorbic acid (AA; also known as vitamin C), through recycling^33^. AA, a broad-spectrum antioxidant, exists in two redox states: reduced AA (rAA) and oxidized (DHA). It provides neuroprotection against reactive oxygen species generated, for example, from high oxidative metabolism in neurons^36^. Reactive oxygen species constantly oxidize rAA to DHA, which is then reconverted back into rAA through astrocytes. The rAA is released into the extracellular space and re-enters neurons for its protective function. Another way to recycle DHA is through efficient intracellular mechanisms of DHA reduction, such as GSH-dependent DHA reduction^37^. AA is transported into the brain and neurons primarily by the sodium-dependent vitamin C transporter 2 (SVCT2)^38^. In addition to its antioxidant properties, AA serves as a cofactor in the synthesis of neurotransmitters like DA and norepinephrine and regulates TH mRNA and protein levels, as shown both in mammalian cell culture models and embryonic brain from transgenic models of the SVTC2 gene^39,40^. In vivo, mDA neurons receive primary input from the striatum, particularly the globus pallidus externa, the primary output for the iSPNs, and the ventral pallidum. In turn, the mDA neurons project to the striatum^41^. This knowledge led to the hypothesis that increased levels of *Gsto2* in iSPNs can disrupt AA homeostasis, leading to changes in TH protein levels and increased DA synthesis in striatal projecting mDA neurons that potentially express no or low TH in the adult brain. To test this hypothesis, we used HPLC to analyse the AA concentration in the striatal and midbrain tissues of both mutants and controls at 3 months and evaluate its impact on the mDA area, specifically the SNc–VTA. Notably, there was a significant decrease (7.5%) in AA in striatal tissues but a significant increase (18%) in SNc–VTA tissues in mutants compared with controls (Fig. 5g). The SVCT2 transporter exhibited behaviour similar to that of AA. Specifically, studies have demonstrated that a functional SVCT2 is glycosylated^42^, causing it to shift from a 70 kDa native form to a higher molecular weight. Remarkably, we discovered a significant increase in the active glycosylated form in the SNc–VTA region of mutants at 3 months and a decrease in the striatal region (Fig. 5h,i), mirroring the decrease in AA concentration in the striatal region and its increase in the mDA area. These results suggest that AA is preferentially taken up by striatal projecting DA neuron terminals, including those possibly expressing low or no TH, through functional SVCT2 and transported in the SNc–VTA region. Depleting BDNF-TrkB signalling in iSPNs increases GSTO2, leading to AA imbalance and dopaminergic dysfunction by increasing TH-expressing cells.

### EBF3 neurons expressing low TH switch to increased TH in mutants

Next, to corroborate these results, we asked about the nature of the newly TH-expressing cells in the mDA area. *Trkb*^*Penk*-KO^ mutants exhibited a significant increase in TH^+^ cells in the SNc–VTA regions after 1 month of GSTO2 upregulation. We deduced that these cells pre-existed in the mDA area and possibly expressed low levels or no TH in adulthood. This feature would make them susceptible to transformation into a dopaminergic phenotype through TH synthesis. Consistent with this hypothesis, recent studies identified various subgroups of mDA neurons through single-cell RNA-seq analysis and established that mDA neuron diversity emerges during postmitotic development. Although most mDA neurons express dopaminergic markers, a few maintain low levels or no TH^43^.

Interestingly, the transcription factor encoding the early B cell factor 3 (*Ebf3*) gene was found to be one of the distinctive markers for the low-TH expressing subgroup among the *Pitx3* isolated mDA neurons. Notably, during early embryonic stages in mice (E13.5 and E15.5), *Ebf3* was broadly expressed. Later, it was mostly enriched in neurons with low TH expression. A similar pattern was observed in human embryos^43^. Therefore, EBF3 and TH co-expressing neurons were identified using EBF3 and TH double immunostaining in the SNc–VTA area of *Trkb*^*Penk*-KO^ mutants and control mice (Fig. 5j–q). Initially, we confirmed the increased TH^+^ cell number in *Trkb*^*Penk*-KO^ mutants (Fig. 5j–n). The number of cells co-expressing EBF3 and TH in the SNc–VTA area was increased in *Trkb*^*Penk*-KO^ mutants, similar to that of TH^+^ cells (Fig. 5o). To ensure that the observed increase in the cells number co-expressing TH and EBF3 was not caused by a general increase in the total number of cells or an increase in the count of cells expressing EBF3, we counted the total number of cells in the SNc–VTA region using DAPI-stained nuclei. No significant difference was observed in the overall cell count between *Trkb*^*Penk*-WT^ and *Trkb*^*Penk*-KO^ mice (Fig. 5p). Similarly, there was no significant difference in the number of EBF3^+^ cells in the SNc–VTA regions between mutant and control mice (Fig. 5q). These results indicate that around 3 months, *Trkb*^*Penk*-KO^ mutants undergo an increase in the number of TH^+^ cells in the SNc–VTA region owing to an increased level of TH expression in EBF3^+^ mDA neurons, which, under physiological conditions, exhibit low levels of TH or no TH.

**Extended Data Fig. 7:**
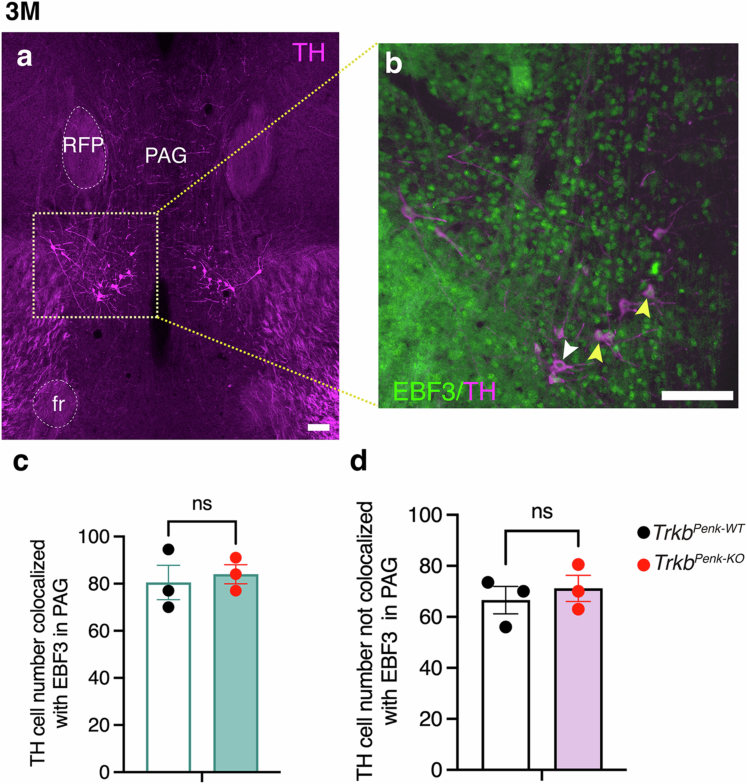
TH+ cell number in the PAG region remains unchanged between mutants and controls. **a**) The representative image from a control mouse shows the PAG in the midbrain region around Bregma −2.488 (based on the Allen Brain Atlas). The image is immunostained for TH and visualised with a 647 nm far-red secondary antibody. Additionally, EBF3 in green (488 nm) is shown together with TH in the inset (**b**). Yellow arrowheads indicate a TH/EBF3 positive colocalised cell and white arrowheads indicate a non-colocalising cell. **c**) Bar plot showing total number of TH+ cells colocalising with EBF3 is similar between mutants and controls (*Trkb*^*Penk-WT*^, 80.5 ± 7.3 vs *Trkb*^*Penk-KO*^, 84 ± 4.0; *p* = 0.7). **d**) Bar plot showing the total number of TH+ positive cells that do not colocalize with EBF3 (*Trkb*^*Penk-WT*^, 66.5 ± 5.3 vs *Trkb*^*Penk-KO*^, 71 ± 5.0; *p* = 0.6). Values are means ± SEM; n = 3 for each genotype/age, male mice. *p* statistic from unpaired, two-tailed, Student’s t-test. Scale bars, **a** and **b**, 100μm; PAG, periacqueductal gray; RFP, retroparafascicular nucleus; fr, fasciculus retroflexus, Related to Fig. 5. Source data

To confirm that the observed phenotype was solely caused by the connection between the striatum and the SNc–VTA region, we used the periaqueductal gray (PAG) region as an internal control. Notably, the PAG region receives no direct afferents from the striatum^44^. As expected, we found no significant difference between the number of TH^+^ cells that colocalized with EBF3 (Extended Data Fig. 7a–c) and those that did not (Extended Data Fig. 7d). These results conclusively support our hypothesis that a specific subset of mDA neurons marked by the *Ebf3* gene typically express little to no TH under physiological conditions but increase TH expression in response to abnormal stimuli.

### Selective *Gsto2* knockdown prevents the early and late onset of motor symptoms in *Trkb*^*Penk*-KO^ mice

**Fig. 6:**
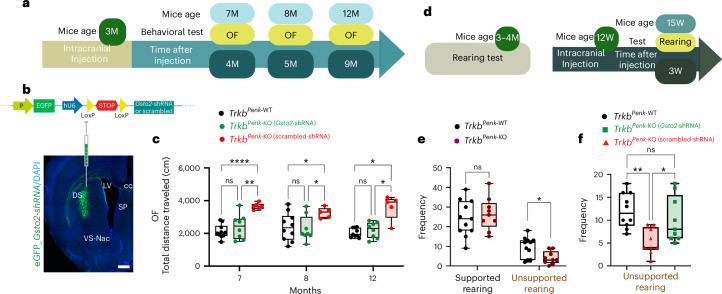
Selective *Gsto2* knockdown prevents the onset of motor symptoms. **a**, Experimental plan layout. Mice were injected either with *Gsto2-*shRNA or scrambled shRNA at 3 months followed by a functional readout. OF, open field. **b**, Graphical view of the viral vector carrying EGFP to highlight successfully transduced cells (as shown in the representative coronal section image), followed by a lox-stop-lox cassette making *Gsto2-*shRNA expressed only in cells carrying Cre recombinase. **c**, Following intracranial injection at 3 months, mice were tested in an OF at 7 months, 8 months and 12 months of age. Data are presented as box plots (boxes show mean and central quartiles; whiskers show data range) and were compared using two-way ANOVA (or mixed model) and Tukey’s multiple comparisons tests. Scrambled injected *Trkb*^*Penk-*KO^ mice developed the hyperlocomotor phenotype as expected (box plots, 7 months, controls vs mutants + scrambled, adj *P* < 0.0001; 8 months, adj *P* = 0.034 and 12 months, adj *P* = 0.018), whereas *Trkb*^*Penk-*KO^ mice injected with *Gsto2*-shRNA were rescued (controls vs mutants + *Gsto2*-shRNA, 7 months, adj *P* = 0.607; 8 months, adj *P* = 0.956 and 12 months, adj *P* = 0.698; mutants + *Gsto2*-shRNA vs mutants + scrambled, 7 months, adj *P* = 0.005; 8 months, adj *P* = 0.025 and 12 months, adj *P* = 0.023). Controls *n* = 9–10; mutant + *Gsto2*-shRNA *n* = 8; mutants + scrambled *n* = 5–6. All female mice. **d**, Experimental plan layout; mice were tested for rearing at 3–4 months. Additional mutant cohorts were injected with either *Gsto2*-shRNA or scrambled shRNA at 3 months and tested 3 weeks later for rearing. W, weeks. **e**, *Trkb*^*Penk*-WT^ *n* = 11, *Trkb*^*Penk-*KO^ *n* = 9 (14 males and 6 females paired) were tested at 3–4 months for supported and unsupported rearing frequency. Data are presented as box plots min–max (median) and compared using unpaired, two-tailed Student’s *t*-test. Unsupported rearing frequency was significantly lower in *Trkb*^*Penk-*KO^ mice (adj *P* = 0.023). **f**, Results presented as box plots min–max (median) and compared using one-way ANOVA are from mice injected at 3 months and tested 3 weeks later for unsupported rearing frequency (controls, *n* = 10 (six males and four females)), mutants injected with scrambled (*n* = 9 (six males and three females)) and mutants injected with *Gsto2*-shRNA (*n* = 9 (five males and four females)). No significant difference in unsupported rearing frequency between mutant injected with *Gsto2*-shRNA and control mice (*F*_(2,25)_ = 7.172, *P* < 0.01; controls vs mutants scrambled shRNA, adj *P* = 0.0029; controls vs mutants *Gsto2*-shRNA, adj *P* = 0.528; mutants scrambled shRNA vs mutants *Gsto2*-shRNA, adj *P* = 0.04). Values in **c**, **e**, and **f** are means; error bars, s.e.m. Scale bar, 500 μm in **b**. Box plot statistics for **c**, **e** and **f** are reported in the figure source data. Source data

**Extended Data Fig. 8:**
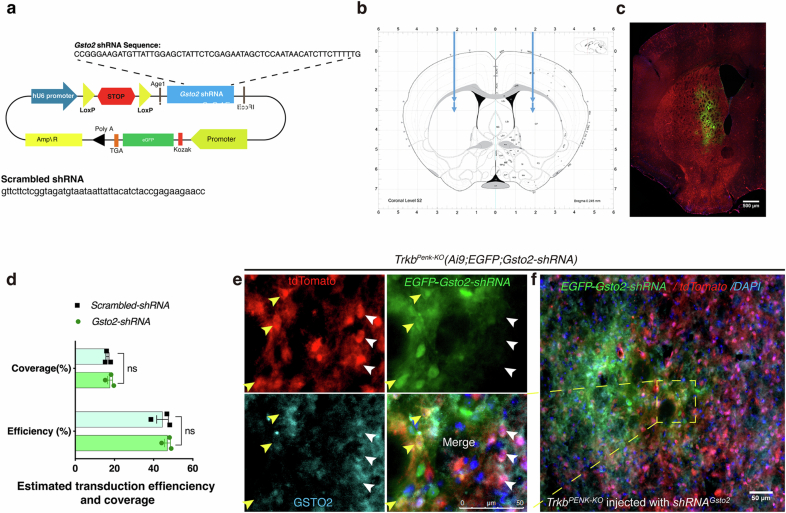
shRNA validation of Cre-dependent recombination. **a**) Graphical view of the viral vector carrying EGFP followed by a lox-stop-lox preceding the *Gsto2*-shRNA or scrambled to allow expression only in cells carrying Cre recombinase. The *Gsto2* and the scrambled sequences are highlighted. **b**) Stereotaxic coordinates were determined from the ‘Mouse Brain in Stereotaxic Coordinates’. A representative coronal section at Bregma 0.245 mm highlights the injection strategy. A maximum of 4 burr holes, 2 in each hemisphere, were used to inject pLKO_EGFP_Lox-STOP-Lox_*Gsto2*-shRNA or scrambled shRNA viral vectors by following specific stereotaxic coordinates. [Anterior-posterior (AP): 0.95 mm; mediolateral (ML): 2.00 mm; dorsoventral (DV): 2.8 and 2.3 mm]. And posterior injection (AP: 0.25 mm; ML: 2.3 mm; DV: 3 and 2.5 mm). To maximise area coverage, we have used two depths x burr hole, injecting 0.625 µl per depth for a total of 1.25 µl per burr hole (details in the Method section). **c**) Representative coronal striatal section of *Gsto2*-shRNA injected *Trkb*^*Penk-KO*^ at Bregma 0.25 mm. **d**) A graph showing the results of viral transduction coverage and efficiency. The percentage of transduced cells compared with the total iSPNs was similar in scrambled shRNA (∼16.4%) and *Gsto2*-shRNA (~17.7%) injected mice. The efficiency of transduced iSPNs was 44.5% for the scrambled shRNA and 47% for the *Gsto2*-shRNA-injected mice. Values are means ± SEM; *p* statistic from unpaired (two-sample t-test). **e**, **f**) Representative *Trkb*^*Penk-KO*^ *Gsto2*-shRNA injected striatal section immunostained for GSTO2 to show the knockdown effect of the shRNA in transduced iSPN^+^ cells (**e**, highlights the yellow area presented in the overview in **f**). tdTomato indicates the enkephalinergic iSPNs, EGFP indicates shRNA transduced cells, and the cyan channel shows the GSTO2 immunostaining. The yellow arrows indicate three iSPNs^+^ transduced with *Gsto2*-shRNA lacking GSTO2 expression, while the white arrows indicate three iSPN^+^ that were not transduced, retaining GSTO2 expression. There were no visible signs of any overt toxic damage in the striatum. Scale bars of images, **c**, 500μm; **e**; **f**, 50μm. Related to Fig. 6. Source data

The above results suggest that disrupted TrkB signalling in iSPNs affects GSH–ascorbate metabolism, particularly GSTO2, which impacts the DA system and thus energy metabolism. Therefore, targeting *Gsto2* in the early stages could prevent the onset of motor symptoms by restoring normal levels of TH and DA. We tested this hypothesis by injecting Cre-dependent *Gsto2*-short hairpin RNA (shRNA) or a scrambled-shRNA viral construct in the striatum of *Trkb*^*Penk*-KO^ mice to allow expression of the shRNA specifically in enkephalinergic neurons at 3 months (Fig. 6a,b and Extended Data Fig. 8a,b). To evaluate the impact of *Gsto2*-specific knockdown, we conducted open field behavioural tests at the ages of 7 months, 8 months and 12 months to assess the blockage of age-related spontaneous motor impairment. Remarkably, scrambled-injected *Trkb*^*Penk*-KO^ mice developed the expected hyperlocomotor phenotype, whereas *Trkb*^*Penk*-KO^ mice injected with *Gsto2*-shRNA showed no significant difference in performance compared to control mice at all ages analysed (Fig. 6c).

It is known that a decrease in rearing is caused by the loss of motor control and an inability to maintain an upright posture^45^. Given that *Trkb*^*Penk*-KO^ mice suffer from subtle deficits in their gait caused by reduced motor control^13^, we first scored mutants and controls at 3–4 months for supported and unsupported rearing. We found a significantly lower frequency of unsupported rearing in *Trkb*^*Penk*-KO^ mice at 3–4 months but no difference in supported rearing frequency (Fig. 6d,e). Next, to assess whether this early motor deficit was also rescued by the downregulation of *Gsto2*, additional cohorts of mutants were injected at 3 months with *Gsto2*-shRNA or scrambled shRNA and tested 3 weeks later for unsupported rearing. As expected, mutants injected with scrambled shRNA showed significantly less unsupported rearing than control mice. Remarkably, there was no significant difference in unsupported rearing frequency between mutants injected with *Gsto2*-shRNA and control mice 3 weeks post treatment (Fig. 6f), suggesting that *Gsto2* upregulation is sufficient to drive the motor dysfunctions in *Trkb*^*Penk*-KO^ mice.

### Molecular rescue by selective in vivo *Gsto2* knockdown

**Fig. 7:**
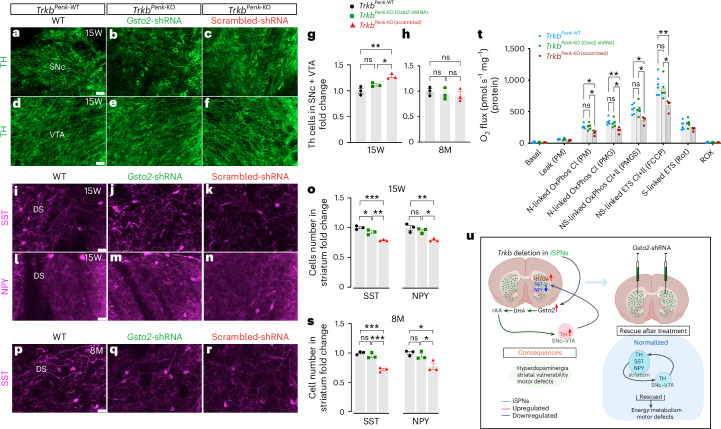
Selective *Gsto2* knockdown rescues the dopaminergic dysfunction and its consequences. **a**–**c**, Representative images from the SNc and **d**–**f** VTA brain regions immunostained with TH from *Trkb*^*Penk-*WT^ control (**a,d**), mutant *Gsto2*-shRNA (**b,e**) or scrambled shRNA (**c,f**). **g**, Scatter–bar plots showing fold change of TH^+^ cell counts in SNc–VTA (significant difference between mutant scrambled shRNA and control mice, adj **P = 0.0029; significant difference between mutant scrambled shRNA and mutant *Gsto2*-shRNA, adj *P = 0.032; no difference between mutant *Gsto2*-shRNA and controls, adj *P* = 0.126 (*n* = 3 each genotype, male mice). **h**, Scatter–bar plots showing analysis of TH^+^ cells in the SNc–VTA region of mutant *Gsto2*-shRNA or scrambled injected at 3 months and analysed at 8 months (TH^+^ cell numbers is similar to control mice). **i**–**k**, Representative striatal images of SST^+^ and **l**–**n**, NPY^+^ immunofluorescence from *Trkb*^*Penk-*WT^ control (**i**,**l**), *Trkb*^*Penk-*KO^ mutants injected at 3 months with *Gsto2*-shRNA (**j**,**m**) or scrambled shRNA (**k**,**n**) in the striatum and analysed 3 weeks later (age 15 W). **o**, Scatter–bar plots showing a significant decrease of striatal immunoreactive SST^+^ and NPY^+^ cells in mutants injected with scrambled shRNA vs control mice, adj ***P = 0.0004 and **P = 0.0052, respectively, a significant difference between mutant scrambled shRNA and mutant *Gsto2*-shRNA, adj **P = 0.0043 and *P = 0.017, respectively; no difference between mutant *Gsto2*-shRNA and control for NPY, adj *P* = 0.5 and an adj *P = 0.046 for SST (*n* = 3 each genotype, male mice). **p**–**r**, Representative striatal images of immunofluorescence for SST from *Trkb*^*Penk-*WT^ control (**p**), *Trkb*^*Penk-*KO^ mutant injected at 3 months with *Gsto2*-shRNA (**q**) or scrambled shRNA (**r**) in the striatum and analysed at 8 months. **s**, Scatter–bar plots showing a significant fold change decrease of striatal immunoreactive SST^+^ and NPY^+^ cells in mutant scrambled shRNA vs controls, adj ***P = 0.0003 and adjusted *P = 0.017, respectively; a significant difference between mutant scrambled shRNA and mutant *Gsto2*-shRNA, adj ***P = 0.0007 and adjusted *P = 0.046, respectively; no difference between mutant *Gsto2*-shRNA and control (SST, adjusted *P = 0.36 and NPY, adjusted *P* = 0.7) (*n* = 3 each genotype, male mice). **t**, Scatter–bar plot showing the oxygen flux for different respiratory states evaluated as per (Fig. 4e–g). Significant differences are indicated by asterisks and are detailed in Table S3 (*TrkB*^*Penk-*WT^, *n* = 6; *TrkB*^*Penk*-KO^ *Gsto2*-shRNA, *n* = 5; and *TrkB*^*Penk*-KO^ scrambled shRNA, *n* = 4; male mice). **u,** Cartoon summary illustrating the consequences of *Trkb* deletion in striatal iSPNs. Values are means; error bars, s.e.m. *P* statistic in **g**, **o** and **s** from one-way ANOVA, followed by Tukey’s multiple comparison analysis; in **t,** from two-way ANOVA and Tukey’s multiple comparisons tests. Scale bars in **a**, **d**, **i**, **m** and **q**, 50 μm. This figure was partially created with BioRender.com (basic section showing the striatum in **u**). Source data

**Extended Data Fig. 9:**
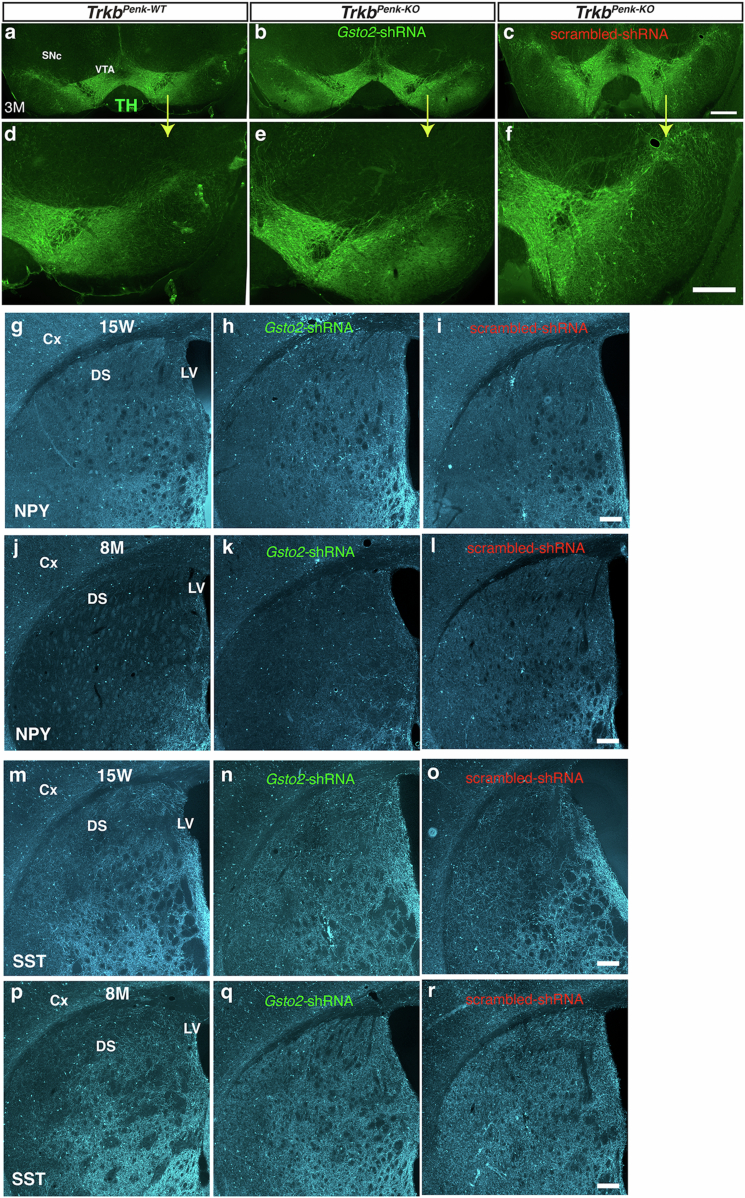
*Gsto2-*shRNA striatal injection rescues the dopaminergic dysfunction and NPY/SST expression. **a**–**c**) Representative images of TH immunofluorescence of the whole SNc/VTA brain region from *Trkb*^*Penk-WT*^ control (**a**), *Trkb*^*Penk-KO*^ mutant injected with *Gsto2*-shRNA (**b**) or scrambled-shRNA (**c**) in the striatum at 3 M. **d**–**f**) shows a higher magnification of one side (as indicated by the arrow) of the SNc/VTA images in panels (**a**–**c**). **g**–**i**) Representative whole dorsal striatal images of NPY immunofluorescence from *Trkb*^*Penk-WT*^ control (**g**), *Trkb*^*Penk-KO*^ mutant injected at 3 M with *Gsto2*-shRNA (**h**) or scrambled-shRNA in the striatum (**i**) and analysed at 15 W. **j**–**l**) Representative whole dorsal striatal images of NPY immunofluorescence from *Trkb*^*Penk-WT*^ control (**j**), *Trkb*^*Penk-KO*^ mutant injected at 3 M with *Gsto2*-shRNA (**k**) or scrambled-shRNA (**l**) in the striatum and analysed at 8 M. **m**–**o**) Representative whole dorsal striatal images of SST immunofluorescence from *Trkb*^*Penk-WT*^ control (**m**), *Trkb*^*Penk-KO*^ mutant injected at 3 M with *Gsto2*-shRNA (**n**) or scrambled-shRNA in the striatum (**o**) and analysed at 15 W. **p**–**r**) Representative whole dorsal striatal images of SST immunofluorescence from *Trkb*^*Penk-WT*^ control (**p**), *Trkb*^*Penk-KO*^ mutant injected at 3 M with *Gsto2*-shRNA (**q**) or scrambled-shRNA (**r**) in the striatum and analysed at 8 M. Scale bars, **c**, 400μm; **f**, 300μm. **i**, **l**, **o** and **r**, 200μm. Related to Fig. 7, the images presented here are from cohorts used in Fig. 7a–f, i–r (n = 3 for each genotype, male mice). These experiments were repeated on additional cohorts, yielding similar results.

Next, to understand the causal relationship between GSTO2 and TH–DA, we assessed whether *Gsto2* downregulation at 3–4 months rescues the TH–DA imbalance and its consequences. Cohorts were injected at 3 months with either *Gsto2*-shRNA or scrambled shRNA (Fig. 6a,b,d experimental design), and their brains were analysed either 3 weeks later (age 15 weeks) or at 8 months (5 months post injection) after behavioural testing. Remarkably, at 15 weeks, there was no significant difference in TH^+^ cell numbers in the SNc–VTA region between *Gsto2*-shRNA-injected mutants and control mice (Fig. 7a,b,d,e,g). By contrast, scrambled-shRNA-injected mutants showed the expected increase in TH^+^ cell numbers compared with control mice and *Gsto2*-shRNA-injected mutants (Fig. 7a–c,d–g and Extended Data Fig. 9a–f). At 8 months, *Gsto2*-shRNA-injected mutants still showed control numbers of TH^+^ cells (Fig. 7h), indicating a sustained rescue of the hyperdopaminergic state.

Furthermore, at 15 weeks, analysis of striatal tissues from *Gsto2*-shRNA-injected mutants showed significant rescue of SST and NPY immunoreactive neuron numbers compared with mutants injected with scrambled shRNA and control mice (Fig. 7i–o and Extended Data Fig. 9g–i,m–o). At 8 months, the results were similar to those obtained at 15 weeks for mutants injected with *Gsto2*-shRNA or scrambled shRNA (Fig. 7p–s and Extended Data Fig. 9j–l,p–r).

We then asked whether normalizing the DA level would improve the energy metabolism deficit. Therefore, striatal tissue from some *Gsto2*-shRNA-injected mutants, controls and mutants injected with scrambled shRNA at 3 months were analysed at 15 weeks. Strikingly, *Gsto2* knockdown rescued the deficit observed at 3 months in diverse respiratory states, as shown in Fig. 7t and Supplementary Table 3. None of the samples had a cytochrome *c* test exceeding 10%, suggesting mitochondrial inner membrane integrity. Together, these results indicate that specific deletion of *Trkb* in iSPNs results in GSTO2 upregulation, which, through its DHAR activity, leads to DA dysfunction in *Trkb*^*Penk*-KO^ mice at the pre-symptomatic stage. This causes striatal vulnerability, affecting energy metabolism and, consequently, motor function (Fig. 7u).

### An HD rat model accurately mimics GSTO2 overexpression

**Fig. 8:**
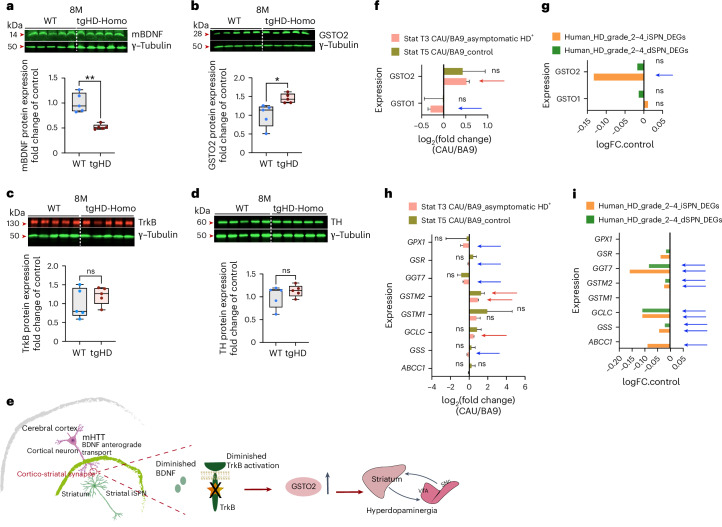
An HD rat model mimics the BDNF decrease and GSTO2 increase preceding TH elevation. **a**–**d**, Representative western blots and box plots (boxes show mean and central quartiles; whiskers show data range) from the rat model SPRDtgHD striatal tissues analysis, *n* = 5 per group (two females and three males) show significantly decreased BDNF, **P = 0.008 (**a**); and increased GSTO2 protein levels, *P = 0.01 (**b**), while TH is still unchanged *P* = 0.33 (**c**) and TrkB levels are similar to controls, *P* = 0.32 (**d**). **e**, Cartoon illustrating the events leading to GSTO2 upregulation by mHTT and its consequences. **f**, Bar plot comparison of gene expression changes in the pre-frontal cortex Brodmann Area 9 (BA9) and the caudate nucleus (CAU) of patients asymptomatic for HD^+^ and control individuals show upregulation of GSTO2 in the CAU brain region of HD^+^ (adj *P* = 1.53 × 10^−15^; red arrow) compared to the non-significant difference in controls (ns, adj *P* = 0.99). GSTO1 is significantly downregulated (blue arrow, adj *P* = 8.67 × 10^−8^) in asymptomatic HD^+^, while non-significantly different (adj *p* = 0.99) in controls. **g**, Bar plot, similar to our results, in patients with grade 2–4 HD, RNA-seq analysis of iSPNs revealed downregulation of GSTO2 (blue arrow, *P* = 3.06 × 10^−6^). **h**, Bar plot showing differential gene expression of some marker genes (as per Fig. 3f) involved in glutathione metabolism (log_2_(fold change) CAU/BA9 of asymptomatic HD^+^ and control brains). Genes indicated with a red arrow were significantly upregulated in asymptomatic HD^+^ (*GCLC*, adj *P* = 3.55 × 10^−21^; *GSTM2* adj *P* = 1.52 × 10^−37^) but not significantly different (ns) in controls apart from *GSTM2* adj *P* = 0.02, whereas genes indicated with a blue arrow were significantly downregulated in asymptomatic HD^+^ (*GPX1*, adj *P* = 0.01; *GSR*, adj *P* = 0.01; *GSS*, adj *P* = 1.72 × 10^−5^; *GGT7*, adj *P* = 1.67 × 10^−17^). No significant changes were observed in the controls. **i**, Bar plot showing differential gene expression of some marker genes (as per Fig. 3f) involved in glutathione metabolism, mostly downregulated or not significantly different in patients with grade 2–4 HD. Values are expressed as log(fold change) control of patients with grade -2–4 HD in iSPN (*ABCC1*, *P* = 6.30 × 10^−5^; *GSS*, *P* = 0.0007; GCLC, *P* = 9.86 × 10^−5^; *GSTM2*, *P* = 0.036; *GGT7*, *P* = 1.49 × 10^−8^); in direct pathway SPN (dSPN) (*GSS*, *P* = 0.04; *GCLC*, *P* = 1.55 × 10^−6^; *GSTM2*, *P* = 0.02; *GGT7*, *P* = 0.0001). Data in **f** and **h** were extracted from a previous publication (additional file 5 (T-test (3) vs (5))^47^; they are from two asymptomatic patients with HD^+^ and two appropriate control brains. Data in **g** and **i** were extracted from a different publication (Table S2)^48^. Values are means; error bars, s.e.m. *P* statistic in **a**–**d** from unpaired, two-tailed Student’s *t*-test. Box plot statistics for **a**–**d** are reported in the figure source data. This figure was partially created with BioRender.com (two neurons in **e**). Source data

The previously unappreciated causal link between altered glutathione–ascorbate metabolism and changes in dopaminergic signalling upon loss of BDNF-TrkB signalling prompted us to investigate its relevance to HD pathogenesis. Post-mortem analysis of brains from patients with HD shows increased striatal and SNc DA and TH activity^3–5^. DA-depleting agents and DA receptor antagonists effectively reduce abnormal movements in HD^3,4^, demonstrating the contribution of DA to motor dysfunction. Although most mouse models of adult-onset HD have not been analysed for changes in DA levels during pre-symptomatic stages^9^, the transgenic rat model (SPRDtgHD) expressing human m*HTT* with 51 CAG repeats^7^ exhibits DA dynamics similar to *Trkb*^*Penk*-KO^ mutants and is reminiscent of human HD. Specifically, they show an early increase in DA neuron counts in the SNc–VTA region and elevated striatal DA levels preceding chorea and non-motor symptoms^46^. Therefore, to validate our results in a suitable rodent model of HD, we used striatal tissues from 8-month-old SPRDtgHD and respective controls. This stage precedes the hyperdopaminergic status observed in this model. BDNF is downregulated in HD brains^10^, reducing TrkB signalling. In SPRDtgHD striatal tissues, as expected, BDNF levels were significantly decreased but not TrkB (Fig. 8a,c). We then measured GSTO2 and TH levels. Remarkably, there was a significant increase in GSTO2 expression before the rise of TH levels, similar to *Trkb*^*Penk*-KO^ mutants. TH levels were still not significantly different from the controls at this stage (Fig. 8b,d). These results suggest that the increased expression of GSTO2 could be the result of reduced BDNF transport in striatal tissue by mHTT followed by increased TH in the SNc–VTA and subsequent striatal hyperdopaminergia (Fig. 8e). This sequence of events is similar to when *Trkb* is removed from striatal iSPNs and supports the idea of GSTO2 being a putative disease-modifying enzyme that could be targeted in the early stages of HD.

### Striatal vulnerability in patients with HD and GSTO2

The cause of neurodegeneration in the striatum of patients with HD is unknown. Characterizing the striatal cells in symptomatic individuals is challenging owing to their massive degeneration. Although rare, the striatum of individuals with asymptomatic HD is mostly intact. A previous study^47^ analysed the gene expression changes in the caudate nucleus (CAU) of two individuals with asymptomatic HD (HD^+^). They then compared these changes with those found in the pre-frontal cortex Brodmann Area 9 (BA9), a relatively unaffected tissue. Studying changes in the less affected tissue (BA9) and the primarily affected brain region (CAU) provided insights into the disease’s progression. Namely, during the prodromal stage of HD in humans, CAU shows significant and distinct alterations in gene expression that include upregulation of GSTO2 compared with the non-significant difference in controls (Fig. 8f). GSTO1 is significantly downregulated in asymptomatic HD^+^ but not in controls (Fig. 8f). Our study also found that *Gsto2* expression decreased with age in *Trkb*^*Penk*-KO^ mice (Fig. 3f and related source data), which is consistent with the results of a previous study^48^ reporting *GSTO2* downregulation in iSPNs in post-mortem analysis of brains from patients with grade 2–4 HD, using RNA-seq analysis (Fig. 8g). In addition, we observed changes in the expression of critical glutathione-related genes in the caudate nucleus of patients who were asymptomatic for HD and iSPNs of patients with grade 2–4 HD (Fig. 8h,i). Overall, these data support our findings in the *Trkb*^*Penk*-KO^ model and the preclinical rat model SPRDtgHD. The similarities indicate that patients with HD may undergo similar changes in glutathione metabolism during the asymptomatic stage owing to the loss of BDNF-TrkB signalling, leading to striatal vulnerability.

## Discussion

This work uncovers a previously unappreciated role for BDNF-TrkB signalling in regulating cellular metabolic pathways, such as glutathione–ascorbate and energy metabolism. Dysfunction in these pathways can trigger neurodegeneration and motor dysfunction. Two key findings reinforce this conclusion. At a pre-symptomatic stage, the deletion of *Trkb* in iSPNs leads to the upregulation of *Gsto2*, increasing DA levels by controlling AA homeostasis and impacting energy metabolism, leading to progressive motor dysfunctions. Selectively reducing *Gsto2* in iSPNs in vivo prevents dopaminergic dysfunction, rescues energy metabolism and halts the development of motor symptoms in *Trkb* mutant mice.

Diverse dysfunctional signalling generally contributes to the onset and progression of neurodegenerative diseases. In rare monogenic disorders such as HD, evidence from patients with HD and rodent models suggests that dysfunctional BDNF-TrkB signalling is involved in the disorder development^10^. It has been hard to understand how it contributes to the development of HD either from post-mortem human brain analysis or from rodent models carrying mHTT, as the latter affects many signalling pathways. To tackle this issue, we started by examining the physiology of BDNF-TrkB signalling in iSPNs, the most vulnerable neurons in HD. TrkB expression is higher in iSPNs than their counterpart, dSPNs^49^, indicating their higher vulnerability to TrkB signalling alteration. We previously demonstrated that specific *Trkb* deletion in iSPNs resulted in age-dependent hyperlocomotor dysfunction, consistent with early hyperkinetic symptoms in HD. This suggests that TrkB signalling deficiency may significantly contribute to HD motor symptoms, as it is essential to maintain normal locomotor behaviour^13^. Using this model, we now demonstrate that *Trkb* deletion-induced iSPN dysfunction leads to a hyperdopaminergic state in the pre-symptomatic phase, before motor dysfunction onset, reminiscent of the dopaminergic dysfunction observed in the brains of patients with HD^3–5^.

Interestingly, there is evidence for reduced striatal DA levels in both R6/2 and YAC128 mice at later disease stages concomitant with motor abnormalities^50–52^. Yet DA dynamics studies at pre-symptomatic stages are lacking in these models. Meanwhile, the SPRDtgHD rat model, like our model, exhibits increased striatal DA–TH levels caused by an increase in mDA neurons^46^. This phenomenon precedes the choreiform symptoms^53^ and is followed by a decrease in striatal DA–TH at late disease stages^54^, supporting our findings. Furthermore, our previous research has demonstrated the functional significance of elevated striatal DA. Administration of cocaine, a potent DA reuptake inhibitor, in 3-month-old pre-symptomatic *Trkb*^*Penk*-KO^ mice reveals a hyperlocomotive phenotype^13^.

Transcriptomic analysis of purified iSPNs at the pre-symptomatic stage has uncovered the early molecular changes driving their vulnerability in the absence of TrkB signalling. We unexpectedly discovered an upregulation of GSTO2, a critical enzyme in glutathione metabolism, which contributed to an imbalance in the redox system GSH/GSSG, probably also affecting closely linked redox ratios NADH/NAD^+^ and NADPH/NADP^+^ (ref. ^55^), and altered AA homeostasis, leading to DA increase. Combined GSH and AA homeostasis impairment and hyperdopaminergic state in *Trkb*^*Penk*-KO^ mice affects energy metabolism, impacting neuronal viability before the onset of motor symptoms. Interestingly, a significant striatal hypometabolism has been observed in patients who are pre-symptomatic for HD, using well-established 18F-FDG PET/CT methods^56^. Metabolic impairments like decreased respiratory exchange rates were also found in the SPRDtgHD rat model from an early age^57^. Thus, the in vivo downregulation of *Gsto2* that rescues DA increase, energy metabolism and motor dysfunctions in *Trkb*^*Penk*-KO^ mice is significant and previously unappreciated. It reveals that altered glutathione and AA homeostasis induced by *Trkb* deletion in iSPNs is directly linked to early dopaminergic dysfunction and disrupted energy metabolism followed by age-dependent motor impairment and strongly supports the relevance of our model to elucidate the mechanisms underlying the vulnerability of iSPNs in neurodegenerative conditions.

The GST family has been implicated in various neurodegenerative diseases, in which either function or changes in gene expression levels affect oxidative stress, contributing to disease susceptibility^27^. GSTO polymorphisms are associated with risk and age-at-onset of Alzheimer’s disease and Parkinson’s disease. For example, the *GSTO2* rs156697 minor allele associated with lower expression of *GSTO2* confers Alzheimer’s disease risk in older age (>80 years)^27^. Instead, the GSTO2 rs2297235 ‘AG’ genotype, which leads to increased expression of *GSTO2*, is significantly associated with age-at-onset of spinocerebellar ataxia type 2 (SCA2)^58^. Interestingly, the dominantly inherited SCAs (including SCA1–SCA3 and SCA6–SCA7), like HD, are caused by CAG repeat expansion that translates into a polyQ stretch. Post-mortem analysis of brains from patients affected by SCA6 has shown reduced BDNF. In a mouse model of SCA6, BDNF and TrkB were downregulated at the age-at-onset, and early activation of TrkB-AKT signalling improved symptoms^59^, supporting our previously unappreciated findings whereby early manipulation of TrkB signalling regulated pathways in which GSH metabolism is crucial to halt the degenerative process triggered in iSPNs lacking BDNF-TrkB signalling. In the Q175-HD mouse model, upregulation of the GSTO2 protein has been reported at 6 months, when motor deficits are not fully developed^60^. Although many mouse models of HD impact BDNF-TrkB signalling, there are no studies to support the involvement of GSTO2 in DA changes in these models. Here, we report that the SPRDtgHD rat model at 8 months displayed decreased striatal BDNF levels and a significant increase in GSTO2 levels. These changes occurred before the increase in TH–DA levels, indicating that the loss of BDNF-TrkB signalling and upregulation of GSTO2 happens before the hyperdopaminergic state, as with the *Trkb*^*Penk*-KO^ mice, supporting that GSTO2 increase is more likely a cause of neurodegeneration than a consequence of it. In addition, SPRDtgHD animals over 6 months old, like *Trkb*^*Penk*-KO^ mice, also exhibited a decreased rearing behaviour, especially in the light phase^57^. Therefore, the hyperkinetic and hyperdopaminergic states observed in the SPRDtgHD rat model and *Trkb*^*Penk*-KO^ mice closely resemble the phenotype of patients with HD.

We next asked the question of how GSTO2 upregulation causes dysfunction in the dopaminergic system. AA is involved in several non-oxidant processes, including epigenetic regulation of TH expression and participation in catecholamine synthesis steps, specifically DA and norepinephrine^61^. It is conceivable that GSTO2 overexpression, through its DHAR activity, accelerates the recycling of AA. Neurons innervating the striatum, including dopaminergic neurons from the SNc–VTA regions, take up AA, which, in low-TH-expressing cells, the phenotype can switch to a dopaminergic one through TH synthesis. Such a switch would explain the rapid increase in TH-positive cells in *Trkb*^*Penk*-KO^ SNc–VTA regions 1 month after GSTO2 upregulation. Here, we show that this is the case (Fig. 7t). Interestingly, such a population of low-TH-expressing cells marked by EBF3 exists in the SNc–VTA^43^. Upon appropriate stimulus—for example, increased AA caused by reduced BDNF-TrkB signalling and upregulation of GSTO2 levels in the iSPNs—the *Ebf3* population undergoes neurotransmitter plasticity, increasing TH synthesis. Our results are supported by experiments whereby the infusion of a D2 receptor agonist into the mouse dorsal striatum increased the TH-positive cell number in SNc^62^. In addition, it is known that the absence of BDNF-TrkB signalling in central neurons can alter metabolism. For example, BDNF signalling is essential in regulating energy balance in mice and humans. Lack of BDNF function can cause hyperphagia and obesity^63^. We have previously demonstrated that the lack of TrkB signalling in cholecystokinin (CCK)-GABAergic neurons leads to glucocorticoid resistance. This results in chronic hypercortisolism, adrenocortical hyperplasia, glucose intolerance and mature-onset obesity, resembling human Cushing’s syndrome^64^. In this report, we present a previously unappreciated finding that BDNF-TrkB signalling regulates glutathione–ascorbate metabolism by modulation of GSTO2 in iSPNs. Additional research is necessary to comprehend the precise mechanism, which may occur by epigenetic regulation of gene expression, either directly or indirectly, through downstream signalling activated by BDNF-TrkB.

Overall, these findings highlight an essential role for BDNF-TrkB signalling in the context of striatal protection and DA circuits (Fig. 7t) and offer a previously unappreciated view for future studies to determine whether pre-symptomatic changes involving the glutathione–ascorbate metabolism pathway, particularly GSTO2, occur in cell types vulnerable to neurodegenerative conditions such as HD or SCAs.

## Methods

### Animals

All animals were kept on a mixed genetic background (C57BL/6J;129). The strains used in this study were *Trkb*^*Penk*-KO^ mice^13^. This line derives from a cross between the *Trkb-*floxed (*Trkb*^lx^) line, as previously described^65^, and the BAC*-Penk*-*cre*^tg/+^ strain, carrying Cre recombinase under the control of the pre-pro-enkephalin promoter as previously described^13^. The *Rosa26-*Ai9*-tdTomato*^21^ reporter line contains a *loxP*-flanked stop cassette before the *tdTomato* cDNA. To highlight the iSPNs, the BAC-*Penk*-Cre line was crossed with the Ai9 reporter line, generating control mice carrying both alleles (BAC-*Penk-cre*^tg/+^; Ai9_ in the presence of an intact *Trkb* allele, called iSPN^+^wt). Crossing the BAC-*Penk-cre*^tg/+^; Ai9 mice with the *Trkb-*floxed line generated the *Trkb*^*Penk*-KO^; Ai9 mutants, called iSPN^+^mut. The age of the mice used in this study ranged from 1 month to 8–12 months. Mice had free access to food and water and were housed in a room with a 12 h light/12 h dark cycle (07:00–19:00 h), ambient temperature at 19–23 °C and humidity at 40–70%. Same-sex mice littermates were group-housed and then used for all different experiments. All experiments were carried out by an experimenter who was blind to genotype, whenever possible. To minimize the impact of bias, we included both male and female mice (mutants and respective gender controls) in the different experiments, as reported in the figure legends.

All animal procedures conformed to the UK legislation Animals (Scientific Procedures) Act 1986 (United Kingdom) and the University of Oxford Ethical Review Committee policy, with a final ethical review by the Animals in Science Regulation Unit (ASRU) of the UK Home Office.

### Histology and immunostaining

Mice were cardiac-perfused with 0.1 M phosphate-buffered saline (PBS, pH 7.4) followed by 4% paraformaldehyde (PFA) in phosphate buffer. Tissues were dissected, post-fixed for 3 h in 4% PFA at room temperature 18–20 ± 2 °C cryoprotected in 30% (w/v) sucrose in Tris-Azide buffer (40 mM Tris, 10 mM phosphate, 0.7% (w/v) NaCl, 0.05% (w/v) NaN_3_) at 4 °C, then embedded in optimal cutting temperature (OCT) compound, rapidly frozen in isopentane on dry ice, and stored at −80 °C until use. Free-floating 30 μm serial brain sections were used for histology and immunofluorescence. For immunofluorescence, sections were washed with PBS at room temperature twice for 10 min, followed by incubation in blocking buffer (5% fish gelatine, 0.5% Triton in PBS) at room temperature for 1 h before incubation with the primary antibodies (see Supplementary Table 4 for primary antibodies used in this study) diluted in the blocking buffer at 4 °C overnight. Afterwards, the sections were washed with Triton X (0.1%) three times (10 min each), followed by incubation with the secondary antibodies conjugated and diluted in the blocking buffer at room temperature for 2 h (see Supplementary Table 4 for secondary antibodies used in this study). After three washes with PBS, sections were incubated with 4’,6-diamidino-2-phenylindol (DAPI, 1:10,000 in PBS) at room temperature for 10 min. Sections were washed three times (15 min each) with PBS before mounting onto the slides. Vectashield mounting medium (Vector Laboratories) was applied on the slide before the coverslip was placed and sealed with nail polish. The slides were dried at room temperature, imaged and stored at −20˚C. All fluorescent images were taken with an upright wide-field epifluorescent microscope from Leica (DM6000B), and images were acquired using a digital Colour Camera (Leica, DFC310 FX) and Leica LAS X software.

### Stereological analysis of striatum and SNc–VTA regions

Brains in OCT blocks from animals (*n* = 3, male and female paired) at different adult ages from each group (unless stated otherwise) were sectioned coronally at 30 μm with Cryostat CM3030 S (Leica Biosystems). For striatal stereological analysis (1.8 mm posterior to bregma 0.345 mm), four sections (one out of every 15) were selected and processed for TH-immunofluorescence, NPY and SST. Striatal TH expression levels were quantified by densitometric analysis using ImageJ. NPY and SST cells were identified based on positive labelling, marked and counted using the Cell Counter plugin of ImageJ (v.1.52p, National Institutes of Health). Image adjustments using ImageJ were necessary to improve the visualization of the target population. Cells were counted twice by an investigator who was blinded to the identity of the samples. Cell numbers are expressed as the mean ± s.e.m., as indicated in the figure legends. For the SNc–VTA analysis (1.32 mm posterior to bregma −2.56 mm), one in every 11 sections (four total) was selected and processed for TH and EBF3 immunofluorescence. TH^+^ and EBF3^+^ cells were counted in the SNc and VTA regions delineated according to the Allen mouse brain atlas. The number of single or colocalized cells, the latter identified with overlapping TH^+^ and EBF3^+^ staining, was performed manually by two independent investigators using the Cell Counter plugin in ImageJ. For the PAG analysis, counts were performed across 0.21 mm postrior to bregma −2.488 mm (two sections were selected, 30 μm in thickness, one in every seven).

### HPLC

#### Tissue preparation

To measure the levels of endogenous DA and related metabolites DOPAC and HVA, striata from adult mice were homogenized in 0.092 M perchloric acid with an ultrasonicator (VibraCell, VCX 500, Sonics) for 10 s. Samples were then centrifuged at 17,949 relative centrifugal force (RCF) at 4 °C for 15 min. Supernatants were analysed for levels of DA, DOPAC and HVA using HPLC with electrochemical detection and normalized to the tissue weight of each sample.

#### HPLC measurement of DA, DOPAC and HVA

DA and metabolites were separated by injecting 50 μl of sample into a Microsorb C18 column (100 × 4.6 mm column, Analytical Column, Waters) and a mobile phase containing 130 mM NaH_2_PO_4_, 2 mM NaCl, 0.1 mM EDTA, 2 mM OSA (1-octanesulphonic acid sodium) and 12.5% (v/v) methanol, pH 3.71, at a flow rate of 1 ml min^−1^. Analytes were electrochemically detected using an amperometric detector (LC-4C, Bio Analytical Systems). The output of the HPLC was analysed using an integrator (Milton Roy, Computing Integrator CI 4000).

#### AA and DHA estimation

AA levels were determined by HPLC using a previously published protocol^66^ with modifications. In brief, the striatal tissues were homogenized using a Dounce homogenizer in a final 20-fold dilution of ice-cold meta-phosphoric acid solution (MPA, 3%) and EDTA (1 mM). The homogenates were centrifuged at 20,817 RCF for 10 min at 4 °C, and the supernatants were collected. The supernatants were divided into two equal volumes to analyse the AA and DHA contents from the same sample to be run on the same day and under similar conditions.

After processing, 10 μl of clean supernatant was injected into the Shimadzu HPLC Prominence System (Shimadzu Corporation). Data were acquired using LabSolutions software (v.5.51). The AA and internal standard (hydroquinone) were resolved using a reverse-phase C18 Atlantis (5 µm, 4.6 mm × 150 mm, Waters Corporation) chromatography column and detected with a UV/Vis detection system (SPD-20A) at 257 nm and 293 nm. The mobile phase used was KH_2_PO_4_ (20 mM, 5.1 pH): methanol with hexadecyl trimethyl ammonium bromide (HTAB, 2 mM) in a 75:25 (v/v) ratio at a flow rate of 1 ml min^−1^ run in isocratic mode. The autosampler (SIL-20AC HT) and the column temperature were maintained at 4 °C and 25 °C, respectively. The total AA, rAA and DHA content were estimated using the subtraction method. rAA is measured in one aliquot of a sample and total AA (that is, rAA and DHA) in a second aliquot of the same sample in which the DHA is reduced by incubation with tris(2-carboxyethyl) phosphine hydrochloride (TCEP, 0.5 mM) in the dark for 30 min at room temperature. Then, the DHA concentration can be indirectly assessed by subtracting the measured rAA concentration from the total AA concentration.

### GSH and GSSG quantification

The tGSH (GSH + GSSG), reduced glutathione and oxidized glutathione (GSSG) levels in the striatal tissues were determined by using a published method^34^ with minor modifications. In brief, the striatum was dissected and snap-frozen in liquid nitrogen and stored at −80 °C until analysis. Immediately before the analysis, the tissues were homogenized using a Dounce homogenizer containing ice-cold homogenization buffer (0.1% Triton X-100 and 0.6% sulfosalicylic acid dissolved in 0.1 M potassium phosphate buffer with 5 mM EDTA disodium salt, pH 7.5). The resulting homogenate was promptly centrifuged at 8,000*g* for 5 min at 2–4 °C to obtain the supernatant used for the GSH and GSSG and protein content estimation. The tGSH concentration was quantified using the enzymatic recycling method in which rates of 5′-thio-2-nitrobenzoic acid (TNB) formation were calculated after the addition of the DTNB (1.8 mM) solution (Ellman’s reagent) and glutathione reductase (250 units per ml) solution to the samples. β-NADPH (0.8 mM) solution was added after 30 s of incubation, and instantly, the absorbance was read at 405 nm every 30 s for 3 min using a microplate reader (LT-4000, LabTech). For GSSG, the samples were treated with 2-vinyl pyridine (2-VP, 0.2% v/v beforehand), which covalently binds with GSH, removing it entirely, thus leaving GSSG as the only measurable substrate of the assay. The excess 2-VP was neutralized with triethanolamine (1% v/v). The final concentrations were determined using linear regression to calculate the values obtained from the standard curves of GSH or GSSG. The GSH concentration was determined by subtracting the GSSG concentration from the tGSH concentration. The concentrations were normalized with protein content determined by BCA assay and are expressed as nmol mg^−1^ of total proteins.

### High-resolution respirometry

Animals aged 3 months and 8 months were killed by cervical dislocation after mild anaesthesia. The striata were quickly dissected on ice and weighed for the wet tissue weight. After weighing, tissues were instantly placed in BIOPS buffer (calcium-EGTA (10 mM), free calcium (0.1 µM), imidazole (20 mM), taurine (20 mM), morpholineethanesulfonic acid (50 mM), dithiothreitol (DTT, 0.5 mM), MgCl_2_ (6.56 mM), ATP 5.77 (mM), phosphocreatine (15 mM); 7.1 pH) and washed with mitochondrial respiration medium MiRO5Cr (EGTA (0.5 mM), MgCl_2_ (3 mM), lactobionic acid (60 mM), taurine (20 mM), potassium dihydrogen phosphate (10 mM), HEPES (20 mM), d-sucrose (110 mM), BSA fatty-acid-free (0.1%) and supplemented with creatine (20 mM) right before use) before homogenization. The homogenates were prepared in MiRO5Cr using a Dounce homogenizer (1 ml, Wheaton Science) at 40–50 strokes. The entire procedure was performed on ice. The resulting homogenates of 1 mg ml^−1^ (2.1 ml) in duplicates were used for respirometry analysis. The remaining homogenate was quickly aliquoted for protein quantification and citrate synthase activity determination and stored at −80 °C until analysis. Tissue homogenates were transferred into calibrated Oxygraph-2k (O2k, Oroboros Instruments), and DatLab software (v.7.4, Oroboros Instruments) was used to record real-time oxygen concentration (µM), as well as oxygen flux per tissue mass (pmol O_2_ s^−1^ mg^−1^). A SUIT protocol (SUIT008) was performed to measure the non-phosphorylating, NADH-linked LEAK-respiration. This was done in the presence of the CI-linked substrates pyruvate (5 mM), malate (2 mM) and in the absence of ADP (2.5 mM) followed by the addition of cytochrome *c* (10 µM) to test for the integrity of the mitochondrial outer membrane (cytochrome *c* control efficiency). Then, glutamate (10 mM) and succinate (10 mM) were added to induce CI and CII substrate-linked respiration. Carbonyl cyanide 4-(trifluoromethoxy) phenylhydrazone (FCCP, 2.5 µM) mediated uncoupling yielded the maximum capacity of the ETS. Rotenone (0.5 µM) was added to inhibit CI to assess succinate-driven ETS capacity. Residual oxygen consumption was measured after the addition of antimycin A (inhibitor of CIII, 2.5 µM) and the values from oxygen flux as a baseline for all respiratory states to obtain mitochondrial respiration. Results were normalized to the protein concentration in the O2k chamber measured and determined by BCA assay (Pierce BCA Protein Assay Kit, Thermo Fisher).

### Immunoblotting

Mice were killed as described above, and the striata were dissected and snap-frozen. Tissues were homogenized in RIPA buffer (Tris (50 mM), NaCl (150 mM), Triton X-100 (1%), disodium EDTA (1 mM), SDS (0.1%), sodium deoxycholate (0.5%)) containing protease and phosphatase inhibitors (S8830, SigmaFast, Thermo Scientific) followed with mild sonication (5 min, cycle: 30 s on/off; Bioruptor). Afterwards, the lysates were centrifuged at 20,000*g* for 20 min at 4 °C and the supernatant was separated. The lysates were treated with loading buffer (protein loading buffer, LI-COR Biosciences) and 100 mM DL-DTT at 80 °C for 4 min. Samples were resolved on an SDS–PAGE gel (10% or 12%) and transferred to nitrocellulose filter membranes using the Bio-Rad apparatus. The protein content was measured using the Pierce BCA Protein Assay Kit. Membranes were blocked in LI-COR Intercept blocking buffer for 1 h at room temperature and incubated with primary antibodies overnight at 4 °C. After washes in PBS with 0.1% Tween (PBS-T) for 30 min (10 min per wash), a secondary antibody was applied for 1 h at room temperature in the blocking buffer (see Supplementary Table 4 for primary and secondary antibodies used in this study). The membranes were washed in PBS-T for 30 min and developed using near-infrared and visible fluorescence with the Odyssey M Imaging system (LI-COR Biosciences), LI-COR acquisition (v.2.2.0.99). Western blots were performed on three animals per group except as otherwise indicated in the figure legend. Results were obtained on two technical replicates, and representative images are shown. Densitometry of immunoreactive bands was performed using the Empiria Studio software (v.2.1.0.134) (LI-COR Biosciences).

For the SPRDtgHD rat tissues, 8-month-old animals were killed via transcardial perfusion with perfusion buffer (0.01 M PBS, pH 7.4). Afterwards, brains were extracted and the regions dissected and snap-frozen in liquid nitrogen for biochemical analysis. Samples were stored at −80 °C until analysed. The procedure for the immunoblotting was the same as that for the mouse tissues described above.

### smFISH

Dissected adult brains (2 months old) were embedded in OCT, frozen and stored at −80 °C until use. smFISH was performed as described by Stellaris RNA FISH frozen tissue protocol (LGC Biosearch Technologies) and a previous publication^67^ with minor modifications. Probe libraries were designed to target the coding sequence of the *Gsto2* gene using the Stellaris Probe Designer online tool. The library consisted of 36 probes of 20 bps each (Supplementary Table 5). The probes were coupled to Quasar 670 fluorophore. Cryosections (8 μm) were collected and directly mounted onto coverslips. Sections were then fixed in 4% PFA for 10 min at room temperature, followed by 1 h permeabilization using 70% ethanol at room temperature. Hybridization with 250 nM fluorescently labelled probes was carried out overnight at 37 °C. Sections were counterstained with DAPI and mounted on slides with Vectashield antifade mounting medium (Vector Laboratories). Image stacks (0.2 μm distance) were acquired with a Leica microscope equipped with an ×100 oil-immersion objective and a Leica DFC 365 FX camera using Leica AF6000 software. Images were processed by 3D reconstruction software (Leica AF6000), followed by image projection. Three to five random fields in the dorsal striatum were imaged for each section (two sections per mouse) for 343 cells for control and 386 cells for mutants analysed from three mice per genotype. The mRNA dots were quantified using the StarSearch tool developed by the Raj Lab (http://rajlab.seas.upenn.edu). DAPI was used to count the number of cells in each field.

### Transcriptome analysis

Bulk RNA-seq was carried out with ~200 sorted adult and aged neurons per replicate. As indicated in the text and figure legends, four to six biological replicates from mutants and controls at two ages (3 months and 8 months, male mice) were used for this experiment. Our previous publication details the brain tissue dissociation, neuronal sorting and profiling methods and the optimized Smart-Seq2 method^20^.

### Bulk RNA-seq and bioinformatics analysis

Bulk RNA-seq samples were sequenced using the TruSeq dual-index sequencing primers on Illumina HiSeq 2000, 2500 or MiSeq (50 bp single-end sequencing) platforms. Sequencing data from pooled lanes were demultiplexed, and after a default quality-filtering step (using FastQC_v.0.10.1), they were recorded in FastQ files representing raw data. Reads were aligned against the murine (mm10) transcriptome (mouse NCBI build37 Refseq transcripts) using Bowtie^68^. Unique reads were counted using featureCounts (v.1.4.5-p1)^69^ and the UCSC mm10 annotation file. All output files were quality assessed using MultiQC (v.0.7)^70^. Non-uniquely mapped reads were discarded. Read counts were then imported into R package DESeq2 (v.1.14.1) for differential gene expression analysis. Counts were normalized using the rlog transformation function in DESeq2 (ref. ^24^) with the blind setting set to true. This log function transforms the data and normalizes gene expression to library size. Reads per kilobase of transcript per million mapped reads (RPKM) values were generated using the EdgeR::rpkm function (v.2.16.5)^71^. Functional analysis of gene expression was performed by using ranked DEGs (adjusted *P* ≤ 0.05) as input into Metacore (v.6.35). GSEA, which allows the detection of modest but coordinated changes in the expression of functionally related groups of genes^32^, was performed using the Liger R package (https://github.com/JEFworks/liger).

### *Gsto2-*shRNA construct

A commercially available lentiviral construct, SIGMA MISSION shRNA encoding plasmid with a verified shRNA targeting *Gsto2* (NM_026619 / pLKO.1) with a mean knockdown level of >90% (ccgggaagatgttattggagctattctcgagaatagctccaataacatcttctttttg) (TRCN0000103074), was modified by adding a STOP cassette just upstream of the shRNA to allow Cre-dependent shRNA expression. A GFP driven by its own promoter is also present in the construct to allow visualization of viral spreading in the brain structure upon intracranial injection. The scrambled-shRNA sequence (gttcttctcggtagatgtaataattattacatctaccgagaagaacc) was taken from https://www.genscript.com/tools/create-scrambled-sequence.

### Surgical procedures

For the treatment with *Gsto2-*shRNA or scrambled shRNA, mutants were randomly selected from ear-notch littermates and assigned to the two experimental groups. Intracranial injections were performed under deep anaesthesia using vaporized isoflurane (2–2.5%) and oxygen (2 l min^−1^). An analgesic cocktail consisting of Metacam (5 mg kg^−1^) and Vetergesic (0.1 mg kg^−1^) was administered by intraperitoneal injection and local injection of Marcain solution (2 mg kg^−1^) underneath the skin of the head. A maximum of four burr holes, two in each hemisphere, were used to inject pLKO_eGFP_Lox-STOP-Lox_Gsto2_shRNA or scrambled-shRNA viral vector with the following stereotaxic coordinates: anterior injection (anterior–posterior (AP): 0.95 mm; mediolateral (ML): 2.00 mm; dorsoventral (DV): 2.8 and 2.3 mm); posterior injection (AP: 0.25 mm; ML: 2.3 mm; DV: 3 and 2.5 mm). The titre of the vector stocks was estimated to be 5.4 × 10^8^ transducing units per ml for the *Gsto2*-shRNA and 5.1 × 10^8^ transducing units per ml for the scrambled shRNA. A total of 5 µl were injected in total (1.25 μl for each burr hole) using a precision 5 μl syringe (Hamilton 75, ESSLAB, cat. no. 7634-01) with a 34 gauge bevelled needle (45^o^) with length 30 mm (Hamilton RN Needle, ESSLAB, cat. no. 207434). To maximize area coverage, we used two depths per burr hole, injecting 0.625 μl per depth for a total of 1.25 μl per burr hole. The viral vector was administered at a rate of 125 nl min^−1^ using an automatic motorized micropump (Ultramicropump3 micro syringe injector with micro4 controller, World Precision Instruments).

### Expression and distribution of injected viral vectors

Immunohistochemistry was used to visualize the distribution of the lentiviral EGFP 3 weeks after injection of the shRNA vector in the striatum of the mice. The coverage of the lentivirus spreading was measured by stereology in a representative set of serial sections. Four sections with an interval of 100 µm centred around the injection site were selected. EGFP-expressing cells were found adjacent to the injection site in the striatum, and most EGFP^+^ cells displayed a neuronal morphology. We calculated the ratio between the number of EGFP^+^ cells and tdTomato^+^ cells (representing enkephalinergic MSNs or iSPN) in striatal sections of both scrambled shRNA and *Gsto2-*shRNA-injected mice brains. The percentage of transduced cells compared with the total iSPNs was similar in scrambled-shRNA (∼16.4%) and *Gsto2-*shRNA (~17.7%) injected mice. The efficiency of transduced iSPNs was calculated by counting cells showing colocalization of both tdTomato and EGFP (44.5% for the scrambled-shRNA and 47% for the *Gsto2-*shRNA) injected mice. No difference was seen in the spread or the transduction efficiency between the scramble and *Gsto2* injected groups. The spread and the efficiency were similar to other striatal lentiviral transductions reported in the literature^72,73^.

### Behavioural analysis

#### Open field

The apparatus consisted of arenas (28 × 28 × 20 cm) with transparent walls and white floors placed within ventilated cabinets (ENV-510, Med Associates), equipped with a dim light and an overhead camera. Locomotor activity, for example, distance travelled (m), was recorded using the Med Associates activity monitor software (v.5.10). Mice were habituated to the arena for 10 min d^−1^ for 3 d (morning sessions). Habituation was evaluated by measuring the distance travelled in the first 5 min. Spontaneous locomotor activity was assessed by recording the distance travelled by mice in 15 min. The experimenter was blind to the genotype of the mice both during the experiment and analysis of data. Arenas were cleaned with 20% ethanol after each trial to minimize olfactory clues, and mice were placed in a new cage after being tested to prevent modifying the behaviour of untested mice.

#### Rearing

Mice were placed into a clear arena (19 cm × 38 cm) with transparent walls (12.5 cm height). After each mouse was habituated to the arena for 5 min, a video camera (1280 × 720 pixel resolution) recorded mouse behaviour in the open field from above at 30 frames per second for 4 min. Rearing was defined as a mouse standing up on its hind limbs while leaning on the wall with one or both paws (supported rearing) or without wall support (unsupported rearing). Supported and unsupported rearing were quantified by an observer blind to the treatment conditions using Behavioural Observation Research Interactive Software (BORIS^74^, v.8.7).

#### Spontaneous spatial novelty preference test

The spontaneous spatial novelty preference test assesses rapidly acquired short-term spatial memory. It is based on the observation that typical mice prefer exploring new spatial environments rather than familiar ones. This test was performed using a Y-maze following a previous publication^75^. Arm allocation (start, other and novel) to specific spatial locations was counterbalanced within each experimental group.

### Statistical analysis

No statistical methods were used to pre-determine sample sizes, but our sample sizes are similar to those reported in previous publications^13,76^ and standards in the field. The number of biological replicates for each experiment is noted in the figure legends. Statistical analysis was performed using GraphPad Prism (v.9). The mean distance travelled in the open field was analysed using multiple *t*-tests, and two-way ANOVA repeated measures were used to analyse habituation to the arena in the open field. Two-way ANOVA was used to analyse the high-resolution respirometry data at 3 months and 8 months. Striatal and SNc–VTA TH, SST, NPY expression and cell count data from each stage were analysed for statistical significance by two-tailed unpaired Student’s *t*-test. All other data, including immunoblots, GSH and AA, were also analysed for statistical significance using a two-tailed unpaired Student’s *t*-test unless stated otherwise. The significance level (alpha) of all tests was set at 0.05, and *P* values were considered significant when *P* < 0.05. More details for statistical tests are indicated in the legend of each figure. Data distribution was assumed normal, but this was not formally tested; however, we show individual data points. Box plot statistical analysis was determined with R software, which uses the shiny package from RStudio (http://shiny.chemgrid.org/boxplotr).

#### Blinding

In experiments for which blinding was feasible (for example, RNA-seq, high-resolution respirometry, behavioural analysis, HPLC and biochemical assays), blinding was implemented using an ID number for the samples and data collection. Additionally, the investigators were unaware of group allocation during the analysis of experimental data. Blinding was not implemented for tissue dissection as this process was not susceptible to collection bias.

### Reporting summary

Further information on research design is available in the Nature Portfolio Reporting Summary linked to this article.

## Supplementary information

Supplementary Information Supplementary Fig. 1, Supplementary Tables 1–5 and Source data for Supplementary Fig. 1, uncropped blots.

Reporting Summary

## Source data

Source Data Fig. 1 Statistical source data.

Source Data Fig. 1 Unprocessed western blots.

Source Data Fig. 2 Statistical source data.

Source Data Fig. 3 Statistical source data.

Source Data Fig. 3 Unprocessed western blots.

Source Data Fig. 4 Statistical source data.

Source Data Fig. 5 Statistical source data.

Source Data Fig. 5 Unprocessed western blots.

Source Data Fig. 6 Statistical source data.

Source Data Fig. 7 Statistical source data.

Source Data Fig. 8 Statistical source data.

Source Data Fig. 8 Unprocessed western blots.

Source Data Extended Data Fig. 1 Statistical source data.

Source Data Extended Data Fig. 2 Statistical source data.

Source Data Extended Data Fig. 4 Statistical source data.

Source Data Extended Data Fig. 5 Statistical source data.

Source Data Extended Data Fig. 6 Statistical source data.

Source Data Extended Data Fig. 6 Unprocessed western blots.

Source Data Extended Data Fig. 7 Statistical source data.

Source Data Extended Data Fig. 8 Statistical source data.

## Data Availability

The paper, extended data, and Supplementary Information contain all the data needed to evaluate this study’s conclusions. The corresponding author provides further details upon reasonable request. Source data are provided with this paper.

## Code availability

RNA-seq data have been deposited in GEO and are available under the accession link https://www.ncbi.nlm.nih.gov/geo/query/acc.cgi?acc=GSE221922. The data in Fig. 8f,h were extracted from a previous publication (their ‘additional file 5 [T-test (3) vs (5)]’)^47^; they are from two asymptomatic patients with HD and two brains from appropriate controls. The data in Fig. 8g,i were extracted from a different publication (their ‘Table S2’)^48^.
