# Supplementary material for: Impaired striatal glutathione–ascorbate metabolism induces transient dopamine increase and motor dysfunction
Source: Nat Metab. 2024 Oct 28;6(11):2100–17. doi: 10.1038/s42255-024-01155-z (PMC11599059; doi:10.1038/s42255-024-01155-z)

nature metabolism

Supplementary information

https://doi.org/10.1038/s42255-024-01155-z

Impaired striatal glutathione–ascorbate metabolism induces transient dopamine increase and motor dysfunction

In the format provided by the authors and unedited

Table of contents

Supplementary Figure 1: *GSTO2 ABS specificity*

Supplementary Table 1: *Selected GSEA pathways from Molecular Signatures Database (MSigDB) curated gene sets 2 (C2) and Hallmark (H) gene sets, affected in iSPN+ depleted of TrkB signalling at 3- and 8M.*

Supplementary Table 2: *Two-way ANOVA results for three relevant respiratory states. Related to Figure 4.*

Supplementary Table 3: *Two-way ANOVA results for relevant respiratory states. Related to Figure 7.*

Supplementary Table 4: *List of primary and secondary antibodies.*

Supplementary Table 5: *Gsto2 smFISH probes library.*

Source Data for Supplementary Figure 1*: Uncropped blots*

Supplementary Figure 1. **GSTO2 ABS specificity**

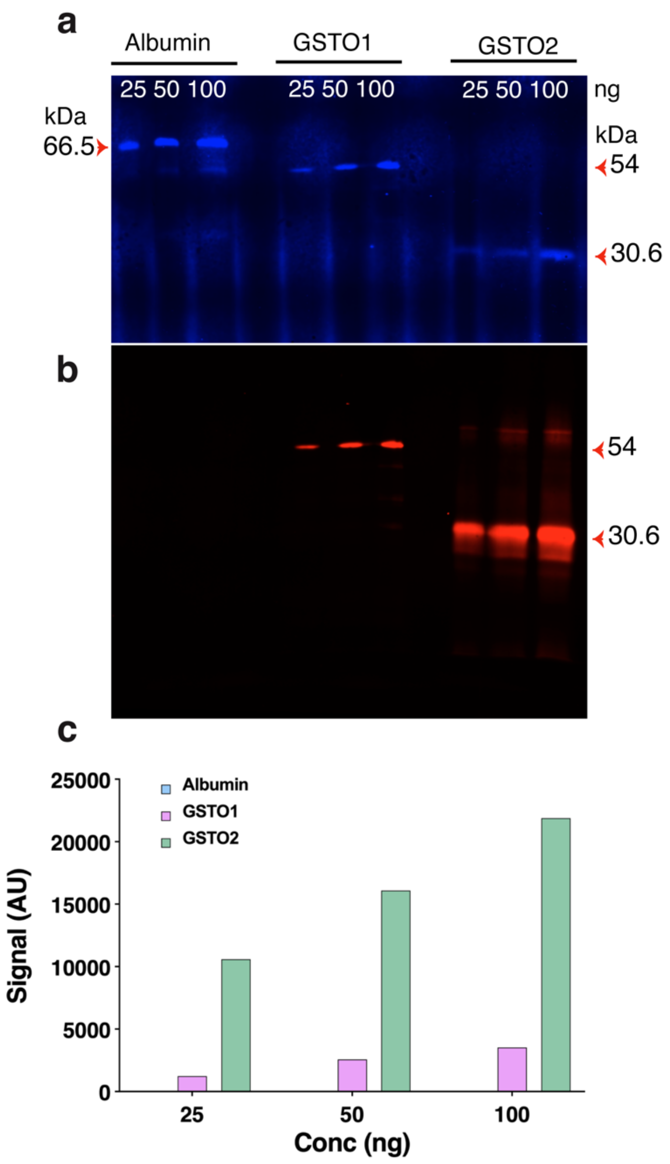

Proteins were resolved on an SDS-PAGE gel (12%) and transferred to a nitrocellulose filter membrane. **a**) Revert total protein stain (Odyssey M Imaging System) was used to confirm the transfer and detection of the proteins on the membrane. About 25, 50 and 100 ng were loaded for each protein. **b**) A representative western blot incubated with GSTO2 primary antibody shows that GSTO2 antibody is highly specific to GSTO2 protein and has negligible cross-reactivity to GSTO1. GSTO1 is an N-terminal GSTtag-GSTO1 recombinant protein, hence appearing as 54kDa. GSTO2 is an N-terminal Histag-GSTO2, hence a molecular weight of 30.6kDa. **c**) The graphical representation of the western blot results shows the high specificity of the signal (expressed in arbitrary units, AU) for GSTO2 compared to GSTO1 and the absence of signal in the albumin lanes. AU, arbitrary units.

Supplementary Table 1. Selected GSEA pathways from Molecular Signatures Database (MSigDB) curated gene sets 2 (C2) and Hallmark (H) gene sets, affected in iSPN+ depleted of TrkB signalling at 3- and 8M.

3M (A to C)

**(A)_GS DETAILS_** MSigDB curated gene sets 2 (C2)_**enriched in iSPN+ mutants at 3 months** compared with iSPN+ controls.	SIZE	ES	NES	NOM p-val	FDR q-val	FWER p-val	RANK AT MAX	LEADING EDGE	Description
REACTOME_GPCR_DOWNSTREAM_SIGNALING	250	0.113	2.07	0.003	0.100	0.995	3646	tags=38%, list=26%, signal=50 %	Genes involved in GPCR downstream signaling
REACTOME_TRANSMISSION_ACROSS_CHEMICAL_SYNAPSES	147	0.130	1.84	0.009	0.20	1	3661	tags=39%, list=27%, signal=53 %	Transmission across Chemical Synapses
REACTOME_SIGNALING_BY_GPCR	315	0.08	1.78	0.008	0.24	1	9335	tags=76%, list=68%, signal=230 %	Signaling by GPCR
REACTOME_GLUTATHIONE_CONJUGATION	15	0.38	1.75	0.016	0.256	1	4925	tags=73%, list=36%, signal=114 %	Genes involved in Glutathione conjugation
KEGG_CALCIUM_SIGNALING_PATHWAY	113	0.14	1.68	0.035	0.286	1	3383	tags=38%, list=25%, signal=50 %	Calcium signalling pathway

**(B)_GS DETAILS_** MSigDB curated gene sets 2 (C2)_**downregulated in iSPN+ mutants at 3 months** compared with iSPN+ controls.	SIZE	ES	NES	NOM p-val	FDR q-val	FWER p-val	RANK AT MAX	LEADING EDGE	Description
REACTOME_RESPIRATORY_ELECTRON_TRANSPORT_ATP_SYNTHESIS_BY_CHEMIOSMOTIC_COUPLING_AND_HEAT_PRODUCTION_BY_UNCOUPLING PROTEINS	75	-0.32	**-3.24**	0	0	0	9012	tags=97%, list=65%, signal=279 %	Genes involved in Respiratory electron transport, ATP synthesis by chemiosmotic coupling, and heat production by uncoupling proteins.
KEGG_OXIDATIVE_PHOSPHORYLATION	102	-0.27	**-3.19**	0	0	0	8688	tags=90%, list=63%,	Genes involved in Oxidative phosphorylation.

								signal=242 %	
REACTOME_TCA_CYCLE_AND_RESPIRATORY_ ELECTRON_TRANSPORT	109	-0.26	-3.21	0	0	0	8159	tags=85%, list=59%, signal=207 %	Genes involved in the citric acid (TCA) cycle and respiratory electron transport.
REACTOME_TRANSLATION	137	-0.31	-4.2	0	0	0	8148	tags=90%, list=59%, signal=217 %	Genes involved in Translation
KEGG_HUNTINGTONS_DISEASE	143	-0.22	-3.05	0	0	0.002	8170	tags=81%, list=59%, signal=197 %	Huntington's disease_genes involved in neuroprotection

**(C)**_MSigDB Hallmark (H) gene sets_ **downregulated in iSPN+ mutants at 3 months** compared with iSPN+ controls.	SIZE	ES	NES	NOM p-val	FDR q-val	FWER p-val	RANK AT MAX	LEADING EDGE	Description
HALLMARK_OXIDATIVE_PHOSPHORYLATION	193	-0.19	-3.2	0	0	0	7579	tags=74%, list=55%, signal=162 %	Genes involved in Oxidative phosphorylation.
HALLMARK_GLYCOLYSIS	154	-0.10	-1.5	0.04	0.12	0.737	9011	tags=76%, list=65%, signal=216 %	Genes encoding proteins involved in glycolysis and gluconeogenesis.
HALLMARK_INFLAMMATORY_RESPONSE	147	-0.10	-1.44	0.090	0.168	0.896	11365	tags=93%, list=82%, signal=518 %	Genes defining inflammatory response.
HALLMARK_TGF_BETA_SIGNALING	51	-0.16	-1.40	0.121	0.179	0.939	8528	tags=78%, list=62%, signal=204 %	Genes upregulated in response to TGFB1

8M (D to F)

**(D)_GS DETAILS_** MSigDB curated gene sets 2 (C2)_**enriched in iSPN+ mutants at 8 months** compared with iSPN+ controls.	SIZE	ES	NES	NOM p-val	FDR q-val	FWER p-val	RANK AT MAX	LEADING EDGE	Description
GRAESSMANN_APOPTOSIS_BY_SERUM_DEPRIVATION_UP	439	0.09	2.23	0.003	0.065	0.84	6344	tags=55%, list=46%, signal=98%	Genes up-regulated in ME-A cells (breast cancer) undergoing apoptosis upon serum starvation (5% to 0% FCS) for 22 hr.
KEGG_APOPTOSIS	76	0.17	1.75	0.01	0.28	1	8218	tags=76%, list=59%, signal=186%	Apoptosis
DUTTA_APOPTOSIS_VIA_NFKB	32	0.26	1.76	0.02	0.27	1	8109	tags=84%, list=58%, signal=202%	NF-kB target genes involved in the regulation of programmed cell death.

**(E)**_MSigDB Hallmark (H) gene sets_ **downregulated in iSPN+ mutants at 8 months** compared with iSPN+ controls.	SIZE	ES	NES	NOM p-val	FDR q-val	FWER p-val	RANK AT MAX	LEADING EDGE	Description
HALLMARK_OXIDATIVE_PHOSPHORYLATION	196	-0.17	**-2.85**	0	0	0	8167	tags=76%, list=59%, signal=182 %	Genes involved in Oxidative phosphorylation.
HALLMARK_GLYCOLYSIS	159	-0.14	**-2.09**	0.001	0.022	0.068	8015	tags=72%, list=58%, signal=168 %	Genes encoding proteins involved in glycolysis and gluconeogenesis.
HALLMARK_REACTIVE_OXIGEN_SPECIES_PATHWA Y	48	-0.19	**-1.61**	0.047	0.169	0.659	8006	tags=77%, list=58%, signal=182 %	Genes upregulated by reactive oxygen species (ROS).

**(F)**_MSigDB curated gene sets 2 (C2)_**downregulated in iSPN+ mutants at 8 months** compared with iSPN+ controls.	SIZE	ES	NES	NOM p-val	FDR q-val	FWER p-val	RANK AT MAX	LEADING EDGE	Description
REACTOME_RESPIRATORY_ELECTRON_TRANSPORT_ATP_SYNTHESIS_BY_CHEMIOSMOTIC_COUPLING_AND_HEAT_PRODUCTION_BY_UNCOUPLING_PROTEINS_	75	-0.33	**-3.44**	0	0	0	5561	tags=73%, list=40%, signal=122%	Genes involved in Respiratory electron transport, ATP synthesis by chemiosmotic coupling, and heat production by uncoupling proteins.
REACTOME_TCA_CYCLE_AND_RESPIRATORY_ELECTRON_TRANSPORT	110	-0.25	**-3.07**	0	3.49E-04	0.002	5561	tags=65%, list=40%, signal=108%	Genes involved in the citric acid (TCA) cycle and respiratory electron transport.

Supplementary Table 2.

Two-way ANOVA results for three relevant respiratory states.

Tukey's multiple comparisons test	Predicted (LS) mean diff.	Summary	Adjusted P Value
**OxPhos CI (PMG)**			
TrkB$^{Penk\text{-}WT}$ 3M vs. *TrkB*$^{Penk\text{-}KO}$ 3M	239.5	*	0.0473
TrkB$^{Penk\text{-}WT}$ 3M vs. *TrkB*$^{Penk\text{-}WT}$ 8M	262.8	*	0.0238
TrkB$^{Penk\text{-}WT}$ 3M vs. *TrkB*$^{Penk\text{-}KO}$ 8M	398.1	****	<0.0001
TrkB$^{Penk\text{-}KO}$ 3M vs. *TrkB*$^{Penk\text{-}WT}$ 8M	23.30	ns	0.9942
TrkB$^{Penk\text{-}KO}$ 3M vs. *TrkB*$^{Penk\text{-}KO}$ 8M	158.6	ns	0.2435
TrkB$^{Penk\text{-}WT}$ 8M vs. *TrkB*$^{Penk\text{-}KO}$ 8M	135.3	ns	0.3822
**OxPhos CI+II (PMGS)**			
TrkB$^{Penk\text{-}WT}$ 3M vs. *TrkB*$^{Penk\text{-}KO}$ 3M	383.8	***	0.0003
TrkB$^{Penk\text{-}WT}$ 3M vs. *TrkB*$^{Penk\text{-}WT}$ 8M	424.3	****	<0.0001
TrkB$^{Penk\text{-}WT}$ 3M vs. *TrkB*$^{Penk\text{-}KO}$ 8M	637.4	****	<0.0001
TrkB$^{Penk\text{-}KO}$ 3M vs. *TrkB*$^{Penk\text{-}WT}$ 8M	40.49	ns	0.9709
TrkB$^{Penk\text{-}KO}$ 3M vs. *TrkB*$^{Penk\text{-}KO}$ 8M	253.6	*	0.0167
TrkB$^{Penk\text{-}WT}$ 8M vs. *TrkB*$^{Penk\text{-}KO}$ 8M	213.1	ns	0.0612
**ETS CI+CII (FCCP)**			
TrkB$^{Penk\text{-}WT}$ 3M vs. *TrkB*$^{Penk\text{-}KO}$ 3M	640.9	****	<0.0001
TrkB$^{Penk\text{-}WT}$ 3M vs. *TrkB*$^{Penk\text{-}WT}$ 8M	695.7	****	<0.0001
TrkB$^{Penk\text{-}WT}$ 3M vs. *TrkB*$^{Penk\text{-}KO}$ 8M	1019	****	<0.0001
TrkB$^{Penk\text{-}KO}$ 3M vs. *TrkB*$^{Penk\text{-}WT}$ 8M	54.73	ns	0.9322
TrkB$^{Penk\text{-}KO}$ 3M vs. *TrkB*$^{Penk\text{-}KO}$ 8M	378.2	****	<0.0001
TrkB$^{Penk\text{-}WT}$ 8M vs. *TrkB*$^{Penk\text{-}KO}$ 8M	323.4	**	0.0011

Related to Figure 4.

Supplementary Table 3

Two-way ANOVA results for relevant respiratory states

Tukey's multiple comparisons test	Mean Diff.	Summary	Adjusted P Value
**N-linked OxPhos CI (PM)**			
TrkB$^{Penk\text{-}WT}$ vs *TrkB*$^{Penk\text{-}KO}$ (*Gsto2*-shRNA)	2.6700	ns	0.9949
TrkB$^{Penk\text{-}WT}$ vs *TrkB*$^{Penk\text{-}KO}$ (Scrambled-shRNA)	99.043	*	0.0116
TrkB$^{Penk\text{-}KO}$ (*Gsto2*-shRNA) vs *TrkB*$^{Penk\text{-}KO}$ (Scrambled-shRNA)	96.373	*	0.0375
**N-linked OxPhos CI (PMG)**			
TrkB$^{Penk\text{-}WT}$ vs *TrkB*$^{Penk\text{-}KO}$ (*Gsto2*-shRNA)	22.972	ns	0.8100
TrkB$^{Penk\text{-}WT}$ vs *TrkB*$^{Penk\text{-}KO}$ (Scrambled-shRNA)	138.27	**	0.0072
TrkB$^{Penk\text{-}KO}$ (*Gsto2*-shRNA) vs *TrkB*$^{Penk\text{-}KO}$ (Scrambled-shRNA)	115.30	*	0.0445
**NS-linked OxPhos CI+II (PMGS)**			
TrkB$^{Penk\text{-}WT}$ vs *TrkB*$^{Penk\text{-}KO}$ (*Gsto2*-shRNA)	-0.94367	ns	0.9998
TrkB$^{Penk\text{-}WT}$ vs *TrkB*$^{Penk\text{-}KO}$ (Scrambled-shRNA)	173.90	*	0.0109
TrkB$^{Penk\text{-}KO}$ (*Gsto2*-shRNA) vs *TrkB*$^{Penk\text{-}KO}$ (Scrambled-shRNA)	174.84	*	0.0421
**NS-linked ETS CI+CII (FCCP)**			
TrkB$^{Penk\text{-}WT}$ vs *TrkB*$^{Penk\text{-}KO}$ (*Gsto2*-shRNA)	54.357	ns	0.8459
TrkB$^{Penk\text{-}WT}$ vs *TrkB*$^{Penk\text{-}KO}$ (Scrambled-shRNA)	322.36	**	0.0085
TrkB$^{Penk\text{-}KO}$ (*Gsto2*-shRNA) vs *TrkB*$^{Penk\text{-}KO}$ (Scrambled-shRNA)	268.00	*	0.0441

Legend: Main respiratory states affected in *TrkB*$^{Penk\text{-}KO}$ (scrambled-shRNA) compared with *TrkB*$^{Penk\text{-}WT}$ controls, for example, N-linked OxPhos CI (PMG), $p = 0.0072$; NS-linked OxPhos CI+II (PMGS), $p = 0.01$; NS-linked ETS CI+CII (FCCP), $p = 0.0085$. However, *Gsto2* knockdown in iSPN by shRNA rescued the mitochondrial defect. *TrkB*$^{Penk\text{-}WT}$ vs *TrkB*$^{Penk\text{-}KO}$ (*Gsto2*-shRNA), N-linked OxPhos CI (PMG), $p = 0.81$; NS-linked OxPhos CI+II (PMGS), $p = 0.99$; NS-linked ETS CI+CII (FCCP), $p = 0.84$. Significant differences are indicated by asterisks and are detailed in the table above. No difference was seen in the basal O_2 consumption rates (mt), leak respiration and residual oxygen consumption (ROX) state ($p>0.05$). *ns,* non-significant. Related to Figure 7.

Supplementary Table 4. List of primary and secondary antibodies.

Primary antibodies

Antibody	Host/type	Dilutions	Supplier and Catalogue number
Enkephalin	Rabbit	1:200/250 (IF)	Neuromics, Ra14124
GAPDH	Rabbit, polyclonal	1:10,000 (WB)	Sigma-Aldrich, G9545
γ-Tubulin	Mouse, monoclonal	1:1500 (WB)	Abcam, ab11316
GSTO2	Rabbit, polyclonal	1:100 (IF)<br>1:500 (WB)	Proteintech, 14562-1-AP
Neuropeptide Y	Rabbit, polyclonal	1:8000 (IF)	Abcam; Ab10980-
Somatostatin	Rabbit, polyclonal	1:1000 (IF)	Atlas; HPA019472
Tyrosine hydroxylase	Mouse, monoclonal	1:500 (IF)<br>1:1000 (WB)	Chemicon, MAB318
BDNF	Rabbit, polyclonal	1:50 (WB)	Santa Cruz, sc-546
TrkB	Rabbit, polyclonal	1:250 (WB)	Cell Signalling, 4603
EBF3	Rabbit, polyclonal	1:1000 (IF)	Sigma-Aldrich, AB10525
SVCT2	Rabbit, polyclonal	1:1000 (WB)	Novusbio, NBP2-13319)

Secondary antibodies

Antibody	Host	Dilutions	Supplier and Catalogue number
Alexa Fluor 488 Anti-mouse	Goat	1:1000 (IF)	Molecular Probes, A-11001
Alexa Fluor 488 Anti-Rabbit	Goat	1:1000 (IF)	Molecular Probes, A-11008
Alexa Fluor 647 Anti-mouse	Donkey	1:1000 (IF)	Molecular Probes, A-31571
IRDye 680RD Anti-Mouse	Goat	1:20,000 (WB)	LI-COR, 925-68072
IRDye 800CW Anti-Rabbit	Goat	1:20,000 (WB)	LI-COR; 925-32211

Supplementary Table 5. *Gsto2* smFISH probes library.

PROBE #	PROBE (5'-> 3')	Probe position	Percent GC
1	ctcacatcctactgggaatc	26	50.00%
2	aatacagcagataaccgccg	109	50.00%
3	gacaggctggatggtaagac	164	55.00%
4	aggtctcacaggcaactact	211	50.00%
5	cactttccactgcagcagat	233	50.00%
6	aacagtcgacagcactcttg	276	50.00%
7	ctccagacccaaactgaatt	300	45.00%
8	taccggaaagctcacagtcg	354	55.00%
9	cggacatcaatctccagtta	383	45.00%
10	tttcccaagcatcttgacaa	408	40.00%
11	catgctgtagattcggatca	463	45.00%
12	tgtgcgagtaggggcagaac	485	60.00%
13	ctttggccttgagaaccagg	512	55.00%
14	ttgatcacttcatgcctgat	534	40.00%
15	aggcttgcttttcaggttaa	556	40.00%
16	ggatgctttgtatagtacca	579	40.00%
17	ctccaagacaggaatttggc	604	50.00%
18	gattcatagaccagctgaca	633	45.00%
19	ccaggtactcacaagcaatg	656	50.00%
20	tacggaaacagctttcttcc	690	45.00%
21	accttacagaatagctccaa	744	40.00%
22	gacattccttgcttaaaggc	767	45.00%
23	cagatccgtacagtctcttc	805	50.00%
24	aatctcttccatgttgcaca	847	40.00%
25	aggtagtgttctgatattca	869	35.00%
26	gatatacagtctccgccgaa	891	50.00%
27	gccagacgaggtaatcaatc	914	50.00%
28	tatacgtccaggcgctcaaa	939	50.00%
29	ggtgtgattcacgcagtcag	967	55.00%
30	ttatcagtgtgcagagcaca	1035	45.00%
31	ttcaagaagcccaggaagac	1059	50.00%
32	agcccaaaatcaaaggcaca	1101	45.00%
33	gcaatgcctggattatcgta	1135	45.00%
34	atcagaatgaccgagagtcc	1158	50.00%
35	caagatgacccgtgatgcag	1180	55.00%
36	cacatccatatttattaggg	1233	35.00%

Source Data for Supplementary Figure 1
Uncropped blots

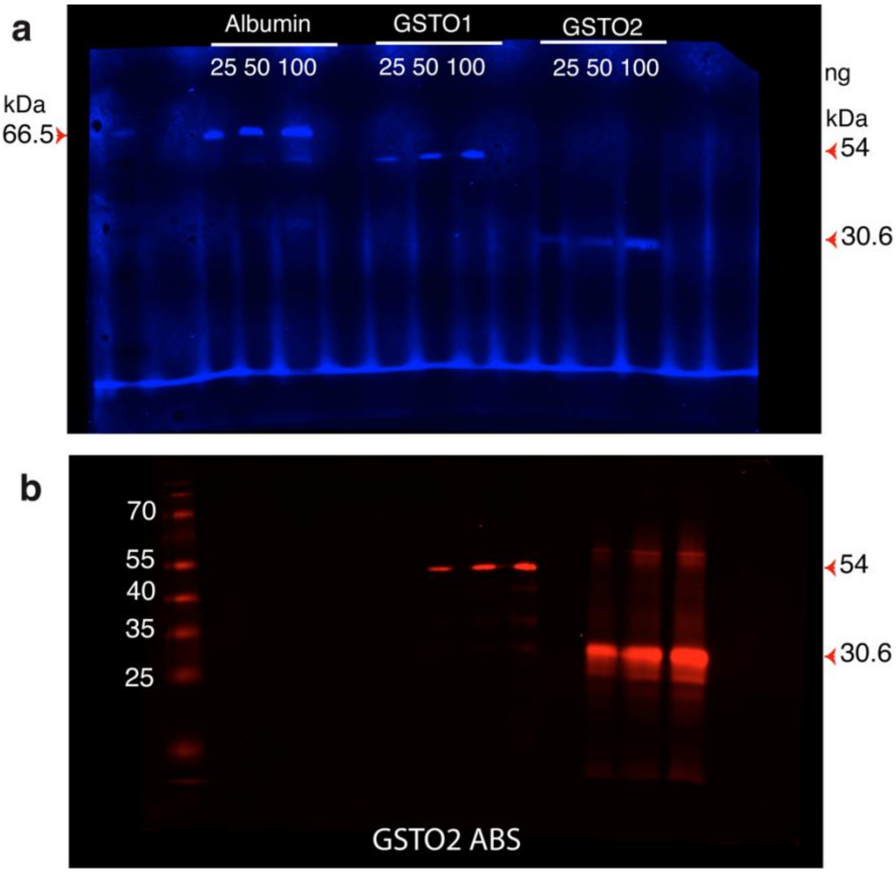

Supplement: Supplementary file 1 — Supplementary Fig. 1, Supplementary Tables 1–5 and Source data for Supplementary Fig. 1, uncropped blots. [file 42255_2024_1155_MOESM1_ESM.pdf]
