# Supplementary material for: Impaired striatal glutathione–ascorbate metabolism induces transient dopamine increase and motor dysfunction
Source: Nat Metab. 2024 Oct 28;6(11):2100–17. doi: 10.1038/s42255-024-01155-z (PMC11599059; doi:10.1038/s42255-024-01155-z)
Supplement: Supplementary file 2 — Reporting Summary [file 42255_2024_1155_MOESM2_ESM.pdf]

nature portfolio

Corresponding author(s): Prof Liliana Minichiello

Last updated by author(s): 06.09.2024

Reporting Summary

Nature Portfolio wishes to improve the reproducibility of the work that we publish. This form provides structure for consistency and transparency in reporting. For further information on Nature Portfolio policies, see our Editorial Policies and the Editorial Policy Checklist.

Statistics

For all statistical analyses, confirm that the following items are present in the figure legend, table legend, main text, or Methods section.

n/a	Confirmed	
☐	☒	The exact sample size (*n*) for each experimental group/condition, given as a discrete number and unit of measurement
☐	☒	A statement on whether measurements were taken from distinct samples or whether the same sample was measured repeatedly
☐	☒	The statistical test(s) used AND whether they are one- or two-sided<br>*Only common tests should be described solely by name; describe more complex techniques in the Methods section.*
☐	☒	A description of all covariates tested
☐	☒	A description of any assumptions or corrections, such as tests of normality and adjustment for multiple comparisons
☐	☒	A full description of the statistical parameters including central tendency (e.g. means) or other basic estimates (e.g. regression coefficient) AND variation (e.g. standard deviation) or associated estimates of uncertainty (e.g. confidence intervals)
☐	☒	For null hypothesis testing, the test statistic (e.g. *F*, *t*, *r*) with confidence intervals, effect sizes, degrees of freedom and *P* value noted<br>*Give P values as exact values whenever suitable.*
☒	☐	For Bayesian analysis, information on the choice of priors and Markov chain Monte Carlo settings
☒	☐	For hierarchical and complex designs, identification of the appropriate level for tests and full reporting of outcomes
☒	☐	Estimates of effect sizes (e.g. Cohen's *d*, Pearson's *r*), indicating how they were calculated

Our web collection on statistics for biologists contains articles on many of the points above.

Software and code

Policy information about availability of computer code

Data collection	• Bulk RNA-seq samples were sequenced using the TruSeq dual-index sequencing primers on Illumina HiSeq 2000, 2500 or MiSeq (50-bp single-end sequencing) platforms. Sequencing data from pooled lanes were demultiplexed, and after a default quality-filtering step (using FastQC_v0.10.1), they were recorded in FastQ files representing raw data.<br>• Leica, LAS X software for image acquisition with Leica epifluorescent microscope.<br>• Datlab software (v 7.4, Oroboros Instruments, Austria) was used to acquire high-resolution respirometry data.<br>• LabSolutions software version 5.51 for HPLC data acquisition.<br>• Immunoblots were imaged using the Odyssey M Imaging system (LI-COR Biosciences, UK), LI-COR acquisition version 2.2.0.99.<br>• Locomotor activity, for example, distance travelled (m), was recorded using the Med Associates activity monitor software version 5.10.
Data analysis	• For the RNAseq data analysis, all output files were quality assessed using MultiQC(v0.7). Reads were aligned against the murine (mm10) transcriptome (mouse NCBI build37 Refseq transcripts) using Bowtie. Unique reads were counted using featureCounts (v1.4.5-p1) and the UCSC mm10 annotation file. All output files were quality assessed using MultiQC (v0.7). Read counts were then imported into R package DESeq2 (v1.14.1) for differential gene expression analysis. Counts were normalised using the rlog transformation function in DESeq2 with the blind setting set to true. Reads per Kilobase of transcript per Million mapped reads (RPKM) values were generated using the EdgeR::rpkm function (v2.16.5). Functional analysis of gene expression was performed by using ranked DEGs (adjusted P≤0.05) as input into Metacore (v6.35) (Thomson Reuters, www.portal.genego.com). Gene set enrichment analysis (GSEA) was performed using the Liger R package (https://github.com/JEFworks/liger).<br>• For the smFISH analysis, the probe libraries were designed to target the coding sequence of the Gsto2 gene using the Stellaris Probe Designer online tool. The mRNA dots were quantified using the StarSearch tool developed by Raj lab (http:/ /rajlab.seas.upenn.edu).<br>• Behavioural Observation Research Interactive Software (BORIS, version 8.7) was used for the rearing analysis.

• ImageJ (vl.52 p, NIH) and Cell Counter Plugin for cell counts and optical density measurements.
• Densitometry analysis of immunoreactive bands was performed using the Empiria Studio software version 2.1.0.134 (LI-COR Biosciences).
• Graph plotting and statistical analysis - Graph Pad Prism v9.

For manuscripts utilizing custom algorithms or software that are central to the research but not yet described in published literature, software must be made available to editors and reviewers. We strongly encourage code deposition in a community repository (e.g. GitHub). See the Nature Portfolio guidelines for submitting code & software for further information.

Data

Policy information about availability of data

All manuscripts must include a data availability statement. This statement should provide the following information, where applicable:

- Accession codes, unique identifiers, or web links for publicly available datasets
- A description of any restrictions on data availability
- For clinical datasets or third party data, please ensure that the statement adheres to our policy

The paper, extended data, and supplementary materials contain all the data needed to evaluate this study's conclusions. The corresponding author provides further details upon reasonable request.
• RNA-sequencing data have been deposited in GEO and are available under the accession link: https://www.ncbi.nlm.nih.gov/geo/query/acc.cgi?acc=GSE221922.
• The data in Figure 8, panels f and h, were extracted from 'additional file 5 [T-test (3) vs (5)]'from Agus et al.,2019, reference47, https://doi.org:10.1186/s12920-019-0581-9; they are from 2 HD+ asymptomatic patients and two appropriate control brains.
• The data in Figure 8, panels g and i were extracted from 'Table S2', from Lee, H. et al. 2020, reference48, https://doi.org:10.1016/j.neuron.2020.06.021.

Research involving human participants, their data, or biological material

Policy information about studies with human participants or human data. See also policy information about sex, gender (identity/presentation), and sexual orientation and race, ethnicity and racism.

Reporting on sex and gender	N/A
Reporting on race, ethnicity, or other socially relevant groupings	N/A
Population characteristics	N/A
Recruitment	N/A
Ethics oversight	N/A

Note that full information on the approval of the study protocol must also be provided in the manuscript.

Field-specific reporting

Please select the one below that is the best fit for your research. If you are not sure, read the appropriate sections before making your selection.

☒ Life sciences ☐ Behavioural & social sciences ☐ Ecological, evolutionary & environmental sciences

For a reference copy of the document with all sections, see nature.com/documents/nr-reporting-summary-flat.pdf

Life sciences study design

All studies must disclose on these points even when the disclosure is negative.

Sample size	No statistical methods were used to pre-determine sample sizes, but our sample sizes are similar to those reported in previous publications (Badurek S. et al, 2020, iScience, 23; Besusso D. et al, 2013, Nat Commun, 4) and standards in the field. The number of biological replicates for each experiment is noted in the figure legends.
Data exclusions	No data were excluded.
Replication	Data presented were replicated in at least two independent experiments. Details are reported in the figure legends as appropriate.
Randomization	Randomization was used were possible. For the treatment with either Gsto2-shRNA or scrambled shRNA, mutants were randomly selected from ear-notch littermates and assigned to the two experimental groups.
Blinding	In experiments where blinding was feasible (for example, RNAseq, high-resolution respirometry, behavioural analysis, HPLC, and biochemical assays), blinding was implemented using an ID number for the samples and data collection. Additionally, the investigators were unaware of group allocation during the analysis of experimental data. Blinding was not implemented for tissue dissection as this process was not

susceptible to collection bias.

Reporting for specific materials, systems and methods

We require information from authors about some types of materials, experimental systems and methods used in many studies. Here, indicate whether each material, system or method listed is relevant to your study. If you are not sure if a list item applies to your research, read the appropriate section before selecting a response.

Materials & experimental systems

n/a	Involved in the study
☐	☒ Antibodies
☒	☐ Eukaryotic cell lines
☒	☐ Palaeontology and archaeology
☐	☒ Animals and other organisms
☒	☐ Clinical data
☒	☐ Dual use research of concern
☒	☐ Plants

Methods

n/a	Involved in the study
☒	☐ ChIP-seq
☒	☐ Flow cytometry
☒	☐ MRI-based neuroimaging

Antibodies

Antibodies used

Antibodies used in the study are detailed in Supplementary Table S4 and reported below as well.
• Enkephalin, Rabbit polyclonal, Neuromics, Cat# Ra14124, used at 1:200/1:250 (IF)
• GAPDH, Rabbit polyclonal, Sigma-Aldrich,Cat# G9545, used at 1:10,000 (WB)
• GSTO2, Rabbit polyclonal, Proteintech, Cat#14562-1-AP, used at 1: 100 (IF)
and 1:500 (WB)
• Neuropeptide Y, Rabbit, polyclonal, Abcam; Cat# ab10980, used at 1:8000 (IF)
• γ-Tubulin, Mouse monoclonal, Abeam, Cat# ab11316, clone GTU-88, used at 1:1500 (WB)
• Tyrosine hydroxylase, Mouse, monoclonal, Chemicon, Cat# MAB318, clone LNCl, used at 1:500 (IF) and 1:1000 (WB)
• Somatostatin, Rabbit polyclonal, Atlas; Cat# HPA019472, used at 1:1000 (IF).
• BDNF, Rabbit, polyclonal lgG, Santa Cruz, (N-20):sc-546, used at 1:50 (WB).
• TrkB (S0E3) Rabbit mAb, Cell Signalling, Cat# 4603, used at 1:250 (WB).
• EBF3, Rabbit, polyclonal, Sigma-Aldrich, AB10525, used at 1:1000 (IF).
• SLC23A2 (SVCT2) Rabbit, polyclonal, NBP2-13319, used at 1:1000 (WB)
• Alexa Fluor 488, goat anti-mouse, Molecular Probes, Cat# A-11001, used at 1:1000 (IF).
• Alexa Fluor 488, goat anti-Rabbit, Molecular Probes, Cat# A-11008, used at 1:1000 (IF).
• Alexa Fluor 647, donkey anti-mouse, Molecular Probes, Cat# A-31571, used at 1:1000 (IF).
• IRDye 680RD, goat anti-Mouse, LI-COR, Cat# 925-68072, used at 1:20, 000 (WB).
• IRDye S00CW, goat anti-Rabbit, LI-COR; Cat# 925-32211 used at 20, 000, (WB).

Validation

These antibodies have been validated for immunostaining or western blot in our previous publications or the company and studies cited on the company's website:
https://www.neuramics.com/RA14124
https://www.sigmaaldrich.com/GB/en/product/sigma/9545
https://www.ptglab.com/products/GST02-Antibody-14562-AP.htm#publications
https://www.citeab.com/antibodies/779134-ab10980-anti-neuropeptide-y-antibody
https://www.abcam.com/gamma-tubulin-antibody-gtu-88-centrosome-marker-ab11316.html
https://www.merckmillipore.com/GB/en/product/Anti-Tyrosine-Hydroxylase-Antibody-clone-LNC1,MM_NF-MAB318?
ReferrerURL=https%3A%2F%2Fwww.google.com%2F
https://www.atlasantibodies.com/products/antibodies/primary-antibodies/trip1e-a-polyclonals/sst-antibody-hpa019472/
https://www.scbt.com/p/bdnf-antibody-n-20#citations
https://www.cellsignal.com/products/primary-antibodies/trkb-80e3-rabbit-mab/4603
https://www.scientificlabs.co.uk/product/antibodies/AB10525
https://www.novusbio.com/products/slc23a2-antibody_nbp2-13319
https:www.thermofisher.com/antibody/product/Goat-anti-Mouse-lgG-H-L-Cross Adsorbed-Secondary-Antibody-Polyclonal/A-11001
https://www.thermofisher.com/antibody/product/Goat-anti-Rabbit-lgG-H-L-Cross-Adsorbed-Secondary-Antibody-Polyclonal/A-11008
https://www.thermofisher.com/antibody/product/Donkey-anti-Mouse-lgG-H-L-Highly-Cross-Adsorbed-Secondary-Antibody-Polyclonal/ A-31571
https://www.licor.com/bio/reagents/irdye-680rd-donkey-anti-mouse-igg-secondary-antibody
https://www.licor.com/bio/reagents/irdye-800cw-goat-anti-rabbit-igg-secondary-antibody

Animals and other research organisms

Policy information about studies involving animals; ARRIVE guidelines recommended for reporting animal research, and Sex and Gender in Research

Laboratory animals

This study used mice of both sexes, ages 1, 2, 3, 5, 8, 10, and 12 months. All animals are kept on a mixed genetic background

Laboratory animals	(C57BL/6J:129).<br>The strains used in this study are:<br>• The BAC-Penk-Cre transgenic line (reference 13, https://doi.org:10.1038/ncomms3031).<br>• Trkb floxed line (reference 65, https://doi.org:10.1016/s0896-6273(00)80853-3).<br>• The Rosa26-tdTomato, Ai9 (reference 21, https://www.nature.com/articles/nn.2467).<br>For the SPRDtgHD rat tissues provided by Prof. Stephan von Horsten (reference 7, https://doi.org:10.1093/hmg/ddg075), the males and females used in this study were of 8M age.
Wild animals	The study did not involve wild animals.
Reporting on sex	None of the findings apply to only one sex. The preliminary studies did not show sex-based differences in the parameters tested in this study; hence, animals from both sexes (paired across the groups) were used unless stated otherwise in the manuscript.
Field-collected samples	The study did not involve field-collected samples.
Ethics oversight	All animal procedures conformed to the UK legislation Animals (Scientific Procedures) Act 1986 (United Kingdom) and the University of Oxford Ethical Review Committee policy, with a final ethical review by the Animals in Science Regulation Unit (ASRU) of the UK Home Office.

Note that full information on the approval of the study protocol must also be provided in the manuscript.

Plants

Seed stocks	N/A
Novel plant genotypes	N/A
Authentication	N/A
