# Supplementary figures and images for: Impaired striatal glutathione–ascorbate metabolism induces transient dopamine increase and motor dysfunction
Source: Nat Metab. 2024 Oct 28;6(11):2100–17. doi: 10.1038/s42255-024-01155-z (PMC11599059; doi:10.1038/s42255-024-01155-z)

Fig 1 (i)

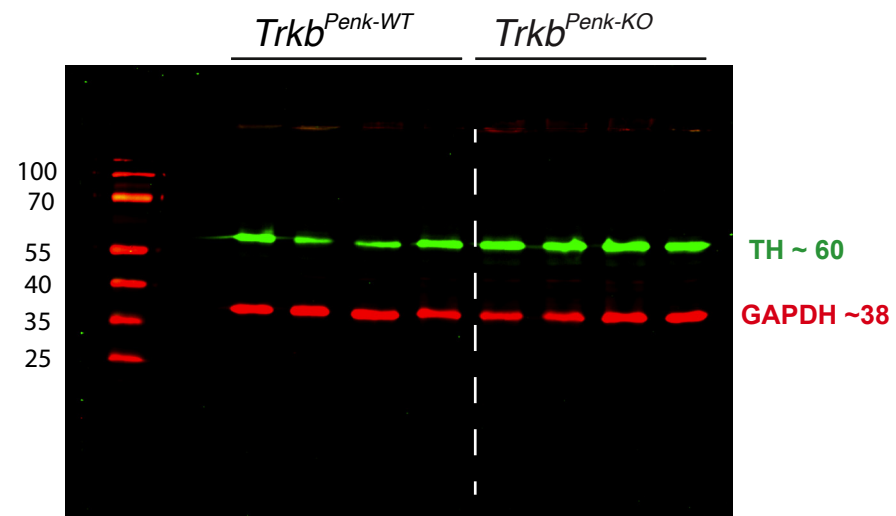

Supplement: Supplementary file 4 — Unprocessed western blots. [file 42255_2024_1155_MOESM4_ESM.pdf]

Fig 3 (g)

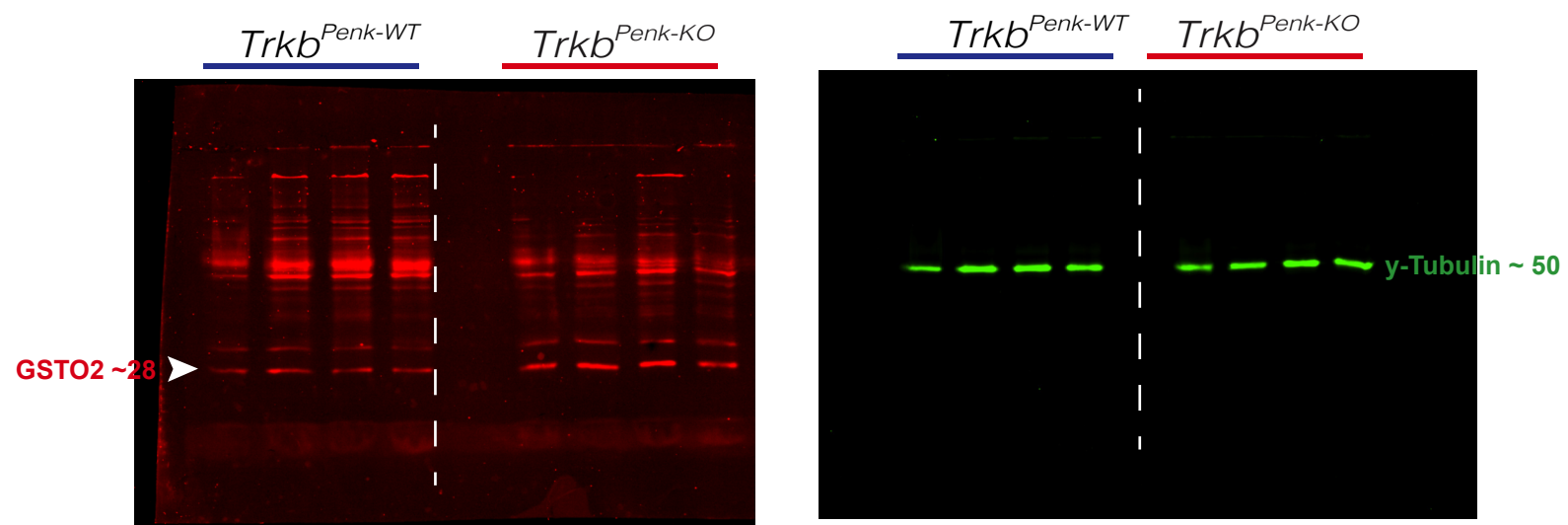

Supplement: Supplementary file 7 — Unprocessed western blots. [file 42255_2024_1155_MOESM7_ESM.pdf]

Fig 5 (f)

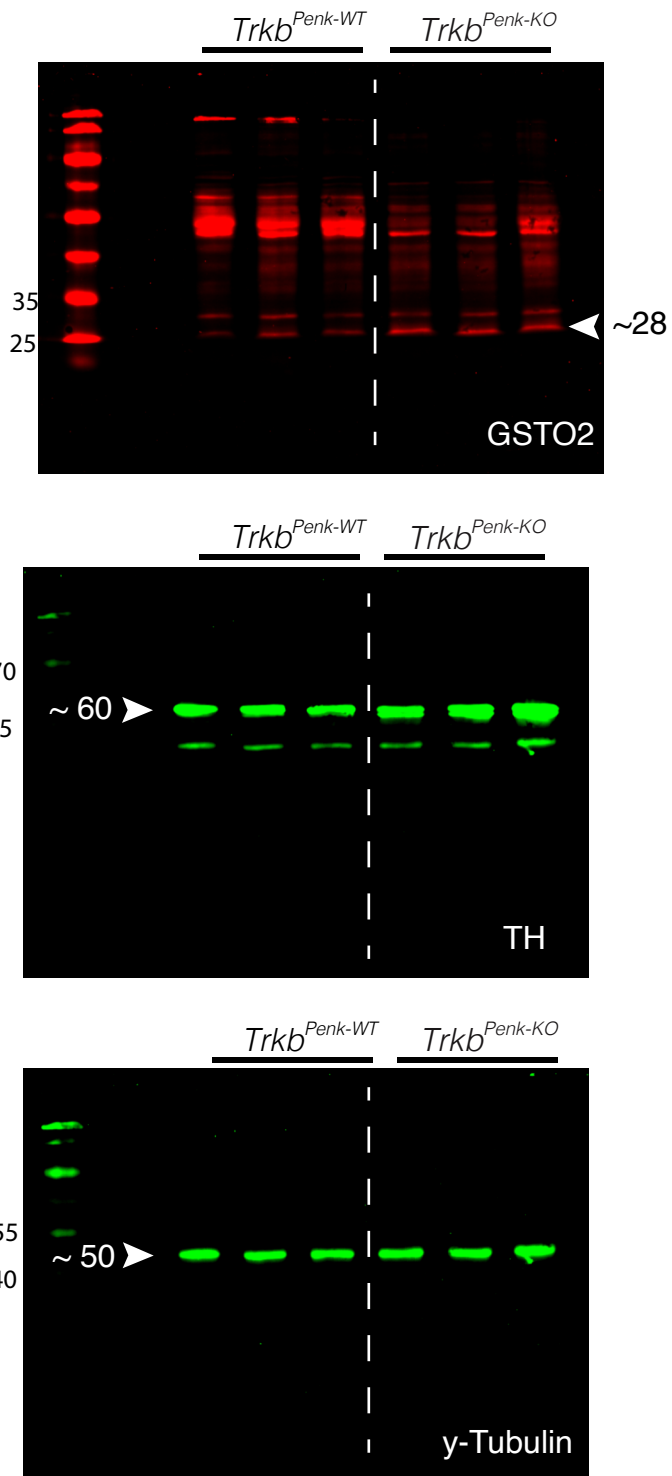

Fig 5 (g)

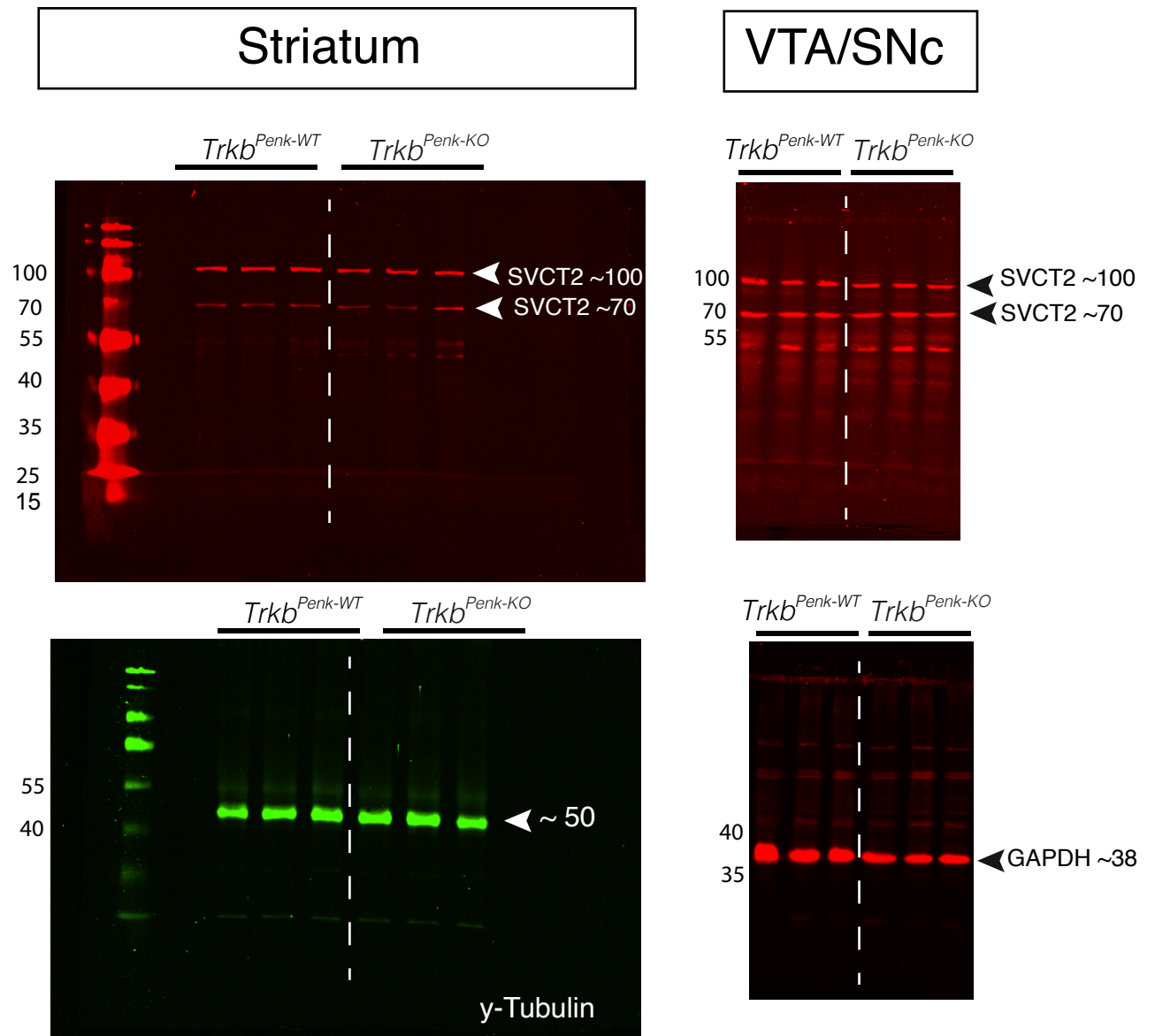

Supplement: Supplementary file 10 — Unprocessed western blots. [file 42255_2024_1155_MOESM10_ESM.pdf]

Fig 8 (a)

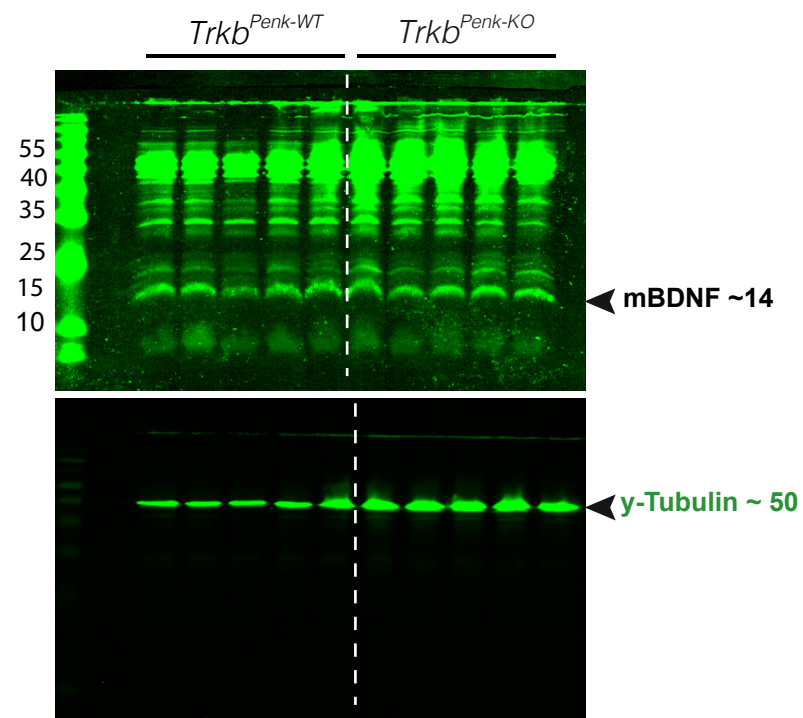

Fig 8 (b)

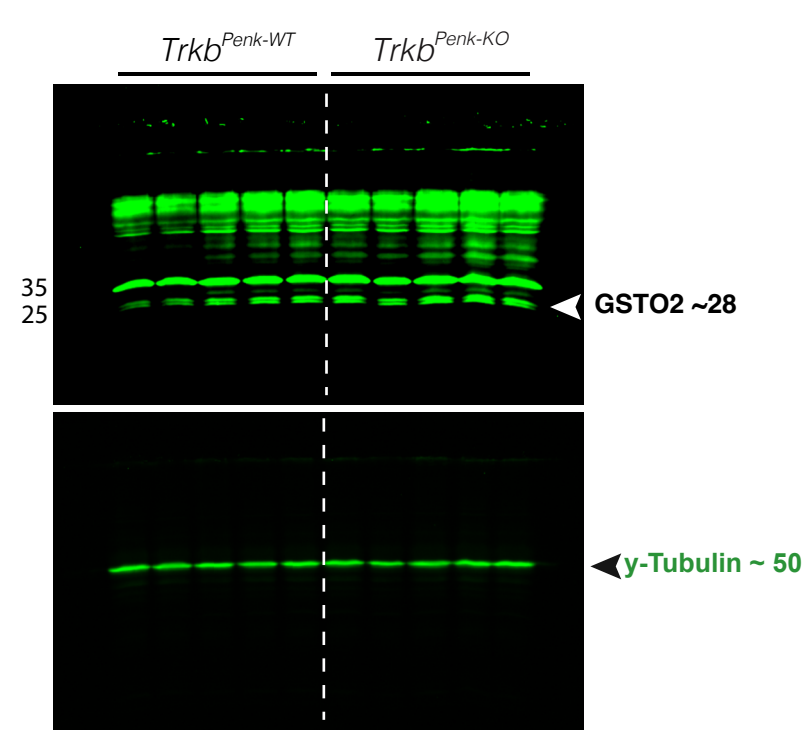

Fig 8 (c)

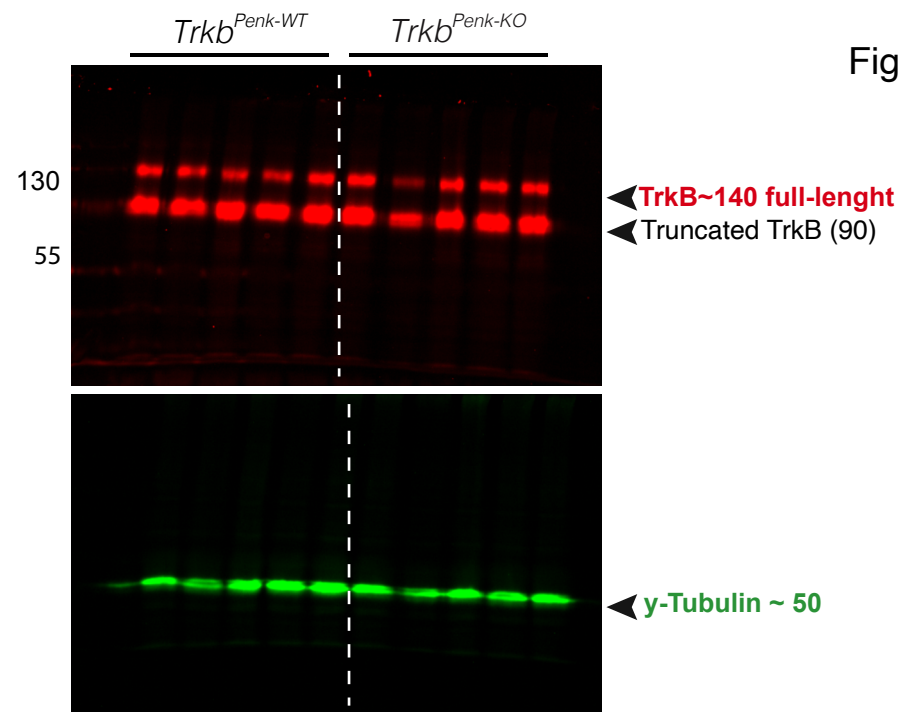

Fig 8 (d)

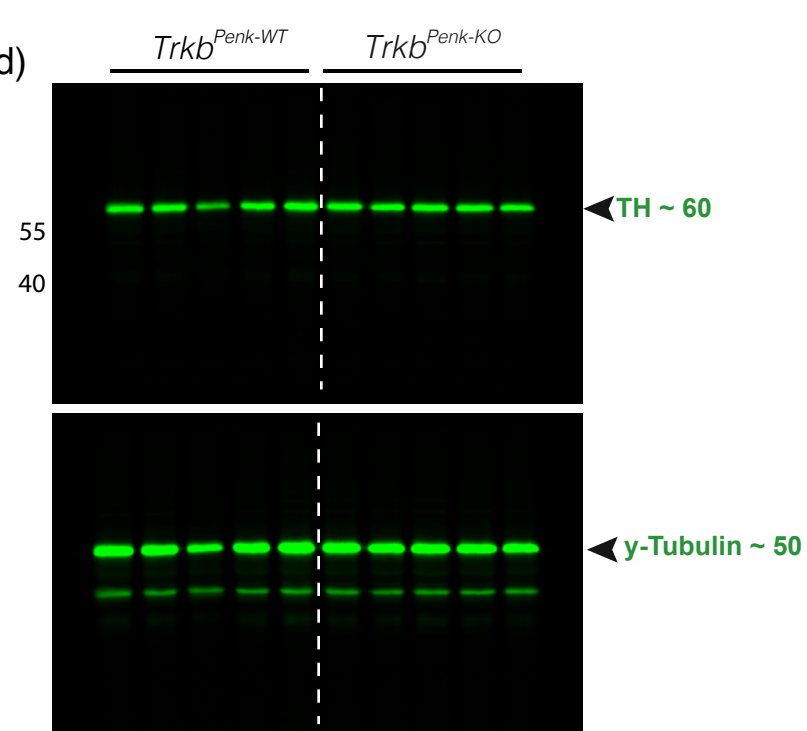

Supplement: Supplementary file 14 — Unprocessed western blots. [file 42255_2024_1155_MOESM14_ESM.pdf]

Extended Data Fig. 6

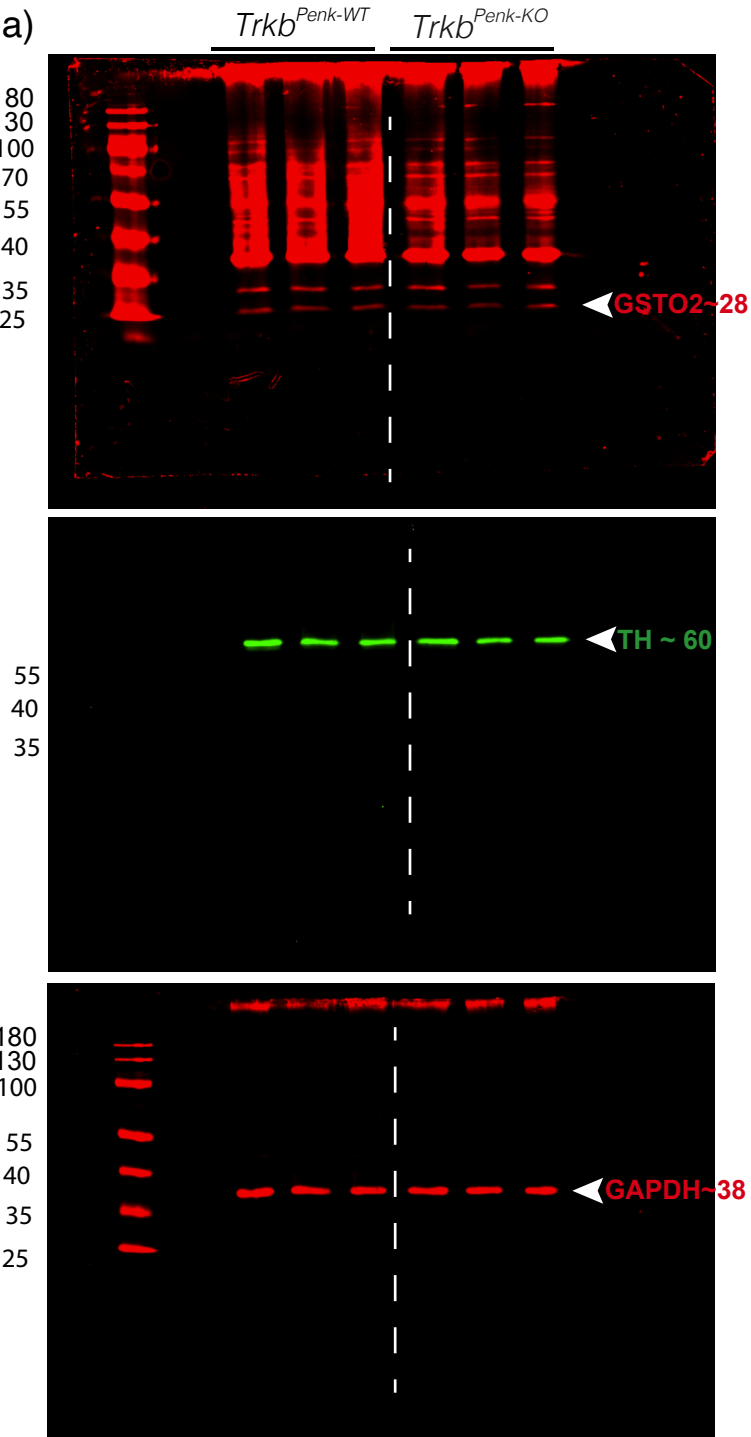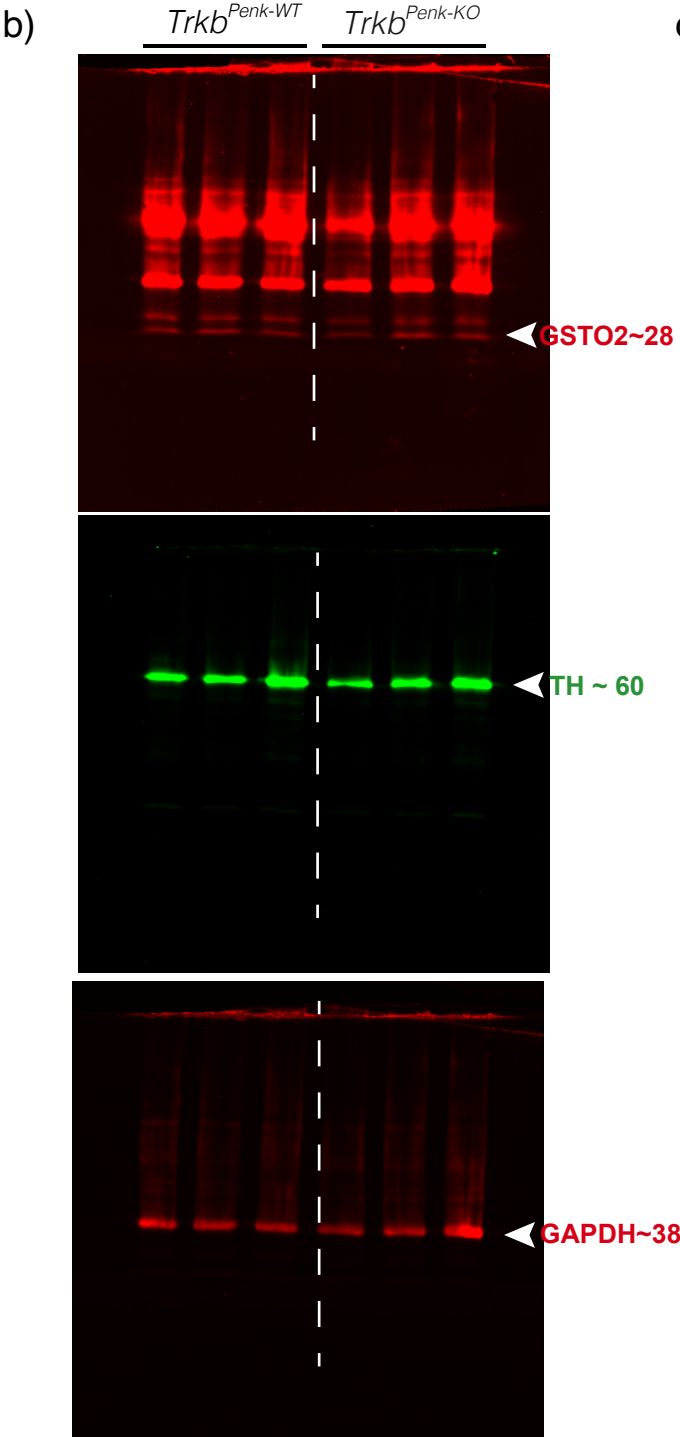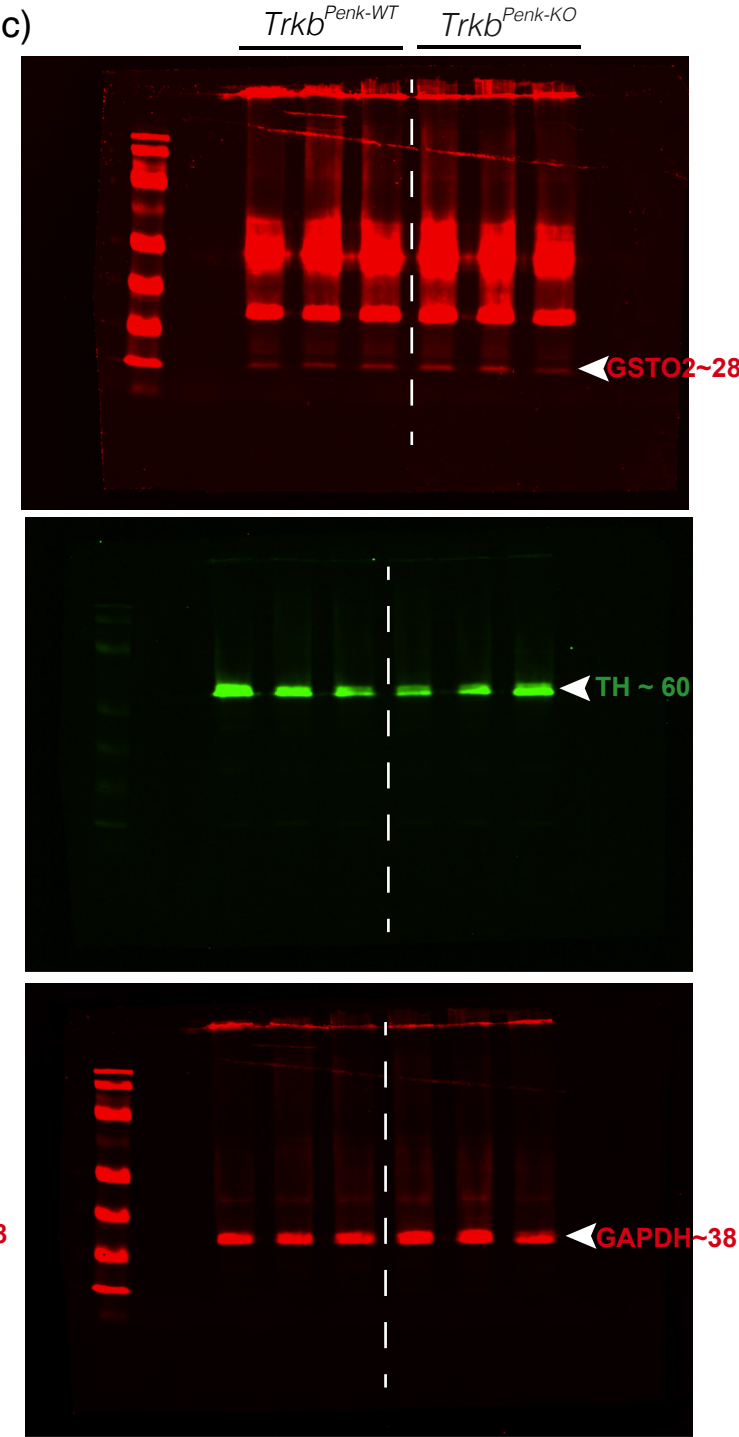

Supplement: Supplementary file 20 — Unprocessed western blots. [file 42255_2024_1155_MOESM20_ESM.pdf]
